# Neferine inhibits BMECs pyroptosis and maintains blood–brain barrier integrity in ischemic stroke by triggering a cascade reaction of PGC-1α

**DOI:** 10.1038/s41598-024-64815-w

**Published:** 2024-06-23

**Authors:** Zi-jian Zheng, Li-zhi Zhu, Han Qiu, Wu-yin-xiao Zheng, Peng-tao You, Shu-he Chen, Chun-ling Hu, Jun-rong Huang, Ya-jun Zhou

**Affiliations:** 1 Department of Pharmacy, Gongan Hospital of Traditional Chinese Medicine, Jingzhou, 434300 China; 2 Hubei University of Chinese Medicine, Wuhan, 430061 China; 3 Department of Pharmacy, The First Affiliated Hospital of Shenzhen University, Shenzhen Second People’s Hospital (Shenzhen Institute of Translational Medicine), Shenzhen, 518055 China; 4 Shenzhen Second People’s Hospital, First Affiliated Hospital of Shenzhen University, 3002 West Sungang Rd, Shenzhen, 518020 China; 5 Department of Pharmacy, Hubei Provincial Hospital of Traditional Chinese Medicine, Wuhan, 430061 China; 6 Hubei Shizhen Laboratory, Wuhan, 430061 China

**Keywords:** Ischemic stroke, Blood–Brain Barrier, Transient middle cerebral artery occlusion, Oxygen and glucose-deprivation/reperfusion, Neferine, PGC-1α, Mitochondria, Inflammasome, Pharmacology

## Abstract

Blood–brain barrier disruption is a critical pathological event in the progression of ischemic stroke (IS). Most studies regarding the therapeutic potential of neferine (Nef) on IS have focused on neuroprotective effect. However, whether Nef attenuates BBB disruption during IS is unclear. We here used mice underwent transient middle cerebral artery occlusion (tMCAO) in vivo and bEnd.3 cells exposed to oxygen–glucose deprivation/reoxygenation (OGD/R) injury in vitro to simulate cerebral ischemia. We showed that Nef reduced neurobehavioral dysfunction and protected brain microvascular endothelial cells and BBB integrity. Molecular docking, short interfering (Si) RNA and plasmid transfection results showed us that PGC-1α was the most binding affinity of biological activity protein for Nef. And verification experiments were showed that Nef upregulated PGC-1α expression to reduce mitochondrial oxidative stress and promote TJ proteins expression, further improves the integrity of BBB in mice. Intriguingly, our study showed that neferine is a natural PGC-1α activator and illustrated the mechanism of specific binding site. Furthermore, we have demonstrated Nef reduced mitochondria oxidative damage and ameliorates endothelial inflammation by inhibiting pyroptosis to improve BBB permeability through triggering a cascade reaction of PGC-1α via regulation of PGC-1α/NLRP3/GSDMD signaling pathway to maintain the integrity of BBB in ischemia/reperfusion injury.

## Introduction

Ischemic stroke is a significant contributor to mortality and disability among adults worldwide. Blood–brain barrier (BBB) plays a critical role in the progression of IS which regulates exchanges of substances between brain and blood. Following the occurrence of IS: it is often accompanied with BBB disruption: and subsequently worse neurological outcomes occurred. BBB integrity is crucial for maintaining the central nervous system homeostasis in IS. Brain microvascular endothelial cells (BMECs): as the mainly physiological structure composition: play a central role in maintaining BBB integrity. Therefore: restoring BMECs function to reestablish integrity of BBB is essential to decrease IS nervous system impairment.

The endogenous protective mechanism of IS involving the ROS detoxification system which plays a crucial role in mitigating the brain injury^1,2^. Peroxisome proliferator-activated receptor γ coactivator 1α (PGC-1α) is the primary factor in antioxidant gene expression^3^. Previous studies have shown that PGC-1α can upgrade superoxide dismutase (SOD) level to reduce oxidative stress damage which induced by cerebral ischemia^4,5^. The core role of reducing oxidative stress during IS highlight the possibility of activating PGC-1α as a new treatment option for IS. In recent years: PGC-1α becoming a target for new drug development: such as Irisin^6^: Songorine^7^.

Neferine (Nef) is a Bisbenzylisoquinoline Alkaloids which extracted from Plumula Nelumbinis. Nef exhibits diverse pharmacological activities: including neuroprotection: anti-platelet aggregation: vasodilation: blood pressure reduction: antioxidant^8–10^. Previous research shows that Nef modulated neurone Nrf2 signalling to protect mitochondrial^11^: diminishing oxidative stress and apoptosis to exert its neuroprotective effect^12^. Research of Nef in IS were focused on neuroprotective effects: the mechanisms of Nef protective effects on BBB remains unclear. Nef has anti-inflammatory effects on LPS-induced endothelial cells injury to prevent atherosclerosis in the early stroke and heart disease^13^. Nef also protects endothelial cell in CKD by blocking the ROS/NLRP3/Capsase-1 signaling pathway^14^. Nef inhibits several inflammatory triggers disease which linked with the aberrant activation of the NOD-like receptor 3 (NLRP3) inflammasome^15,16^. Previous studies have clarified that Nef could inhibits activated NLRP3 inflammasome: but the specific protein target in anti-inflammatory pathway still not identified. And the impact of Nef on vascular endothelium after stroke is also unclear.

We speculate that Nef may maintain the integrity of BBB in IS by protecting BMECs: and the protection effects may be related to signal molecules upstream of NLRP3. To confirm the hypothesis: we employed the transient middle cerebral artery occlusion (tMCAO) in vivo and oxygen glucose deprivation/reperfusion (OGD/R) in vitro to clarify the protective effect of neferine on BBB following ischemia/reperfusion (I/R) injury. It was found that Nef protects BBB by protecting the structure and function of BMECs in vitro and in vivo. In addition: we found that the activation of PGC-1α inhibits mitochondrial oxidative stress damage: reduces mtROS levels: and thus inhibits pyroptosis related pathways: which is a potential mechanism for Nef to improve the blood–brain barrier in ischemic stroke.

## Results

### Nef reduces injury of bEnd.3 cells underwent OGD/R

**Figure 1:**
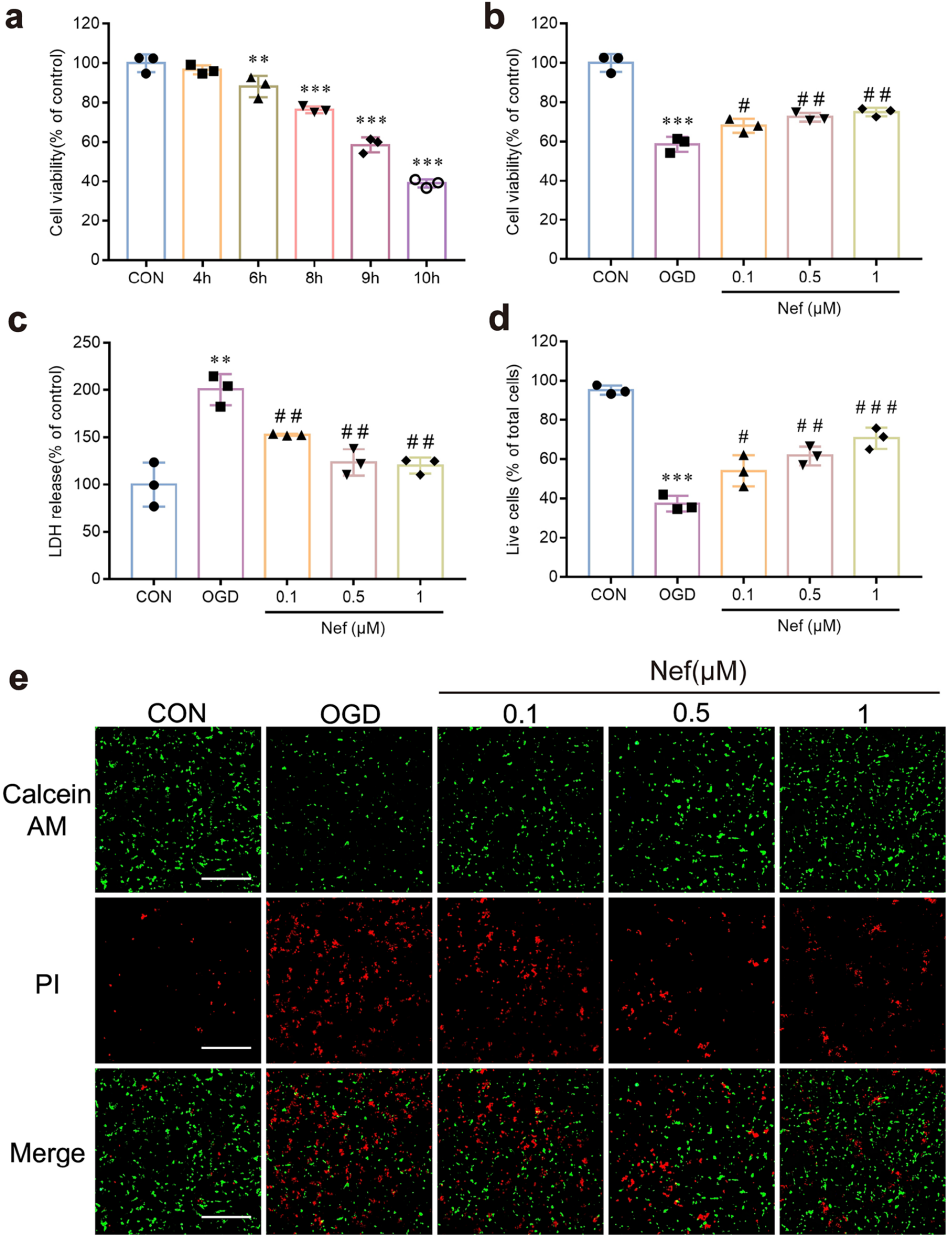
Effects of Nef on the cell viability of bEnd.3 cells under OGD/R conditions. Cell viability were measured after exposed to OGD for 4 to 10 h (**a**). Effect of Nef treatment (0.1 μM: 0.5 μM: 1 μM) on cell viability (**b**). The LDH release was measured after being treated with Nef (0.1 μM: 0.5 μM: 1 μM) during 9 h of OGD by a commercial kit (**c**). The live cells proportion was measured after being treated with Nef (0.1 μM: 0.5 μM: 1 μM) during 9 h of OGD by Live/Dead assay kit (**d**,**e**). scale bar: 200 μm. The experiments were performed in triplicate and repeated at least three times in different days. ^**^P < 0.01: ^***^P < 0.001νs. CON: ^*#*^*P* < 0.05: ^*##*^*P* < 0.01: ^*###*^*P* < 0.001νs. OGD.

To verify the protective effect of Nef on BMECs: we exposed the bEnd.3 cells (BMECs of mouse) to OGD/R injury to mimic BMECs damage during cerebral I/R in vitro. Cells viability was evaluated using CCK-8 assay. As time progressed: cell viability declined (Fig. 1a). In contrast: Nef treatment increased cell viability (0.1: 0.5: and 1 μM) (Fig. 1b). Nef (0.1 μM) has been already markedly reduced the LDH leakage under OGD/R condition: and the effect of reduction is more obvious with increase of the dose (Fig. 1c). The staining images were used to revealed the PI-positive cells (dead cells) (Fig. 1e). Dead cells were evidently higher in OGD/R-exposed groups and Nef reduced the death of the OGD/R-treated bEnd.3 cells (Fig. 1d,e). These data demonstrated that Nef rescued OGD-induced cell death in bEnd.3 cells.

### Nef alleviated brain ischemia–reperfusion induced brain injury and improves the integrity of BBB in mice

**Figure 2:**
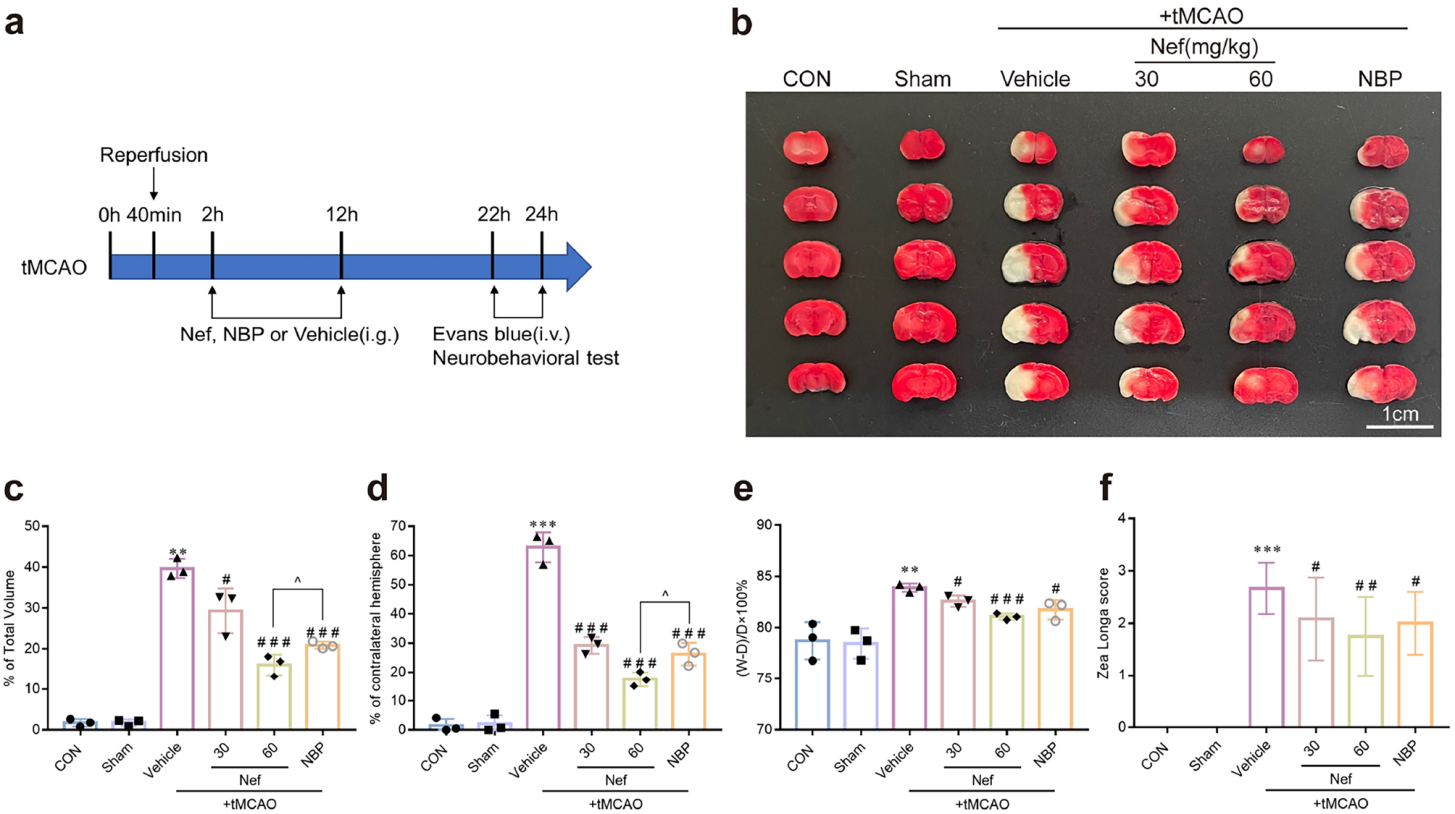
Effects of Nef on cerebral infarct volume: brain swelling volume: brain water content and neuroethology in mice induced by tMCAO. Experimental design of tMCAO model (**a**). Representative photographs of mice brain tissue stained with 0.5% TTC solution (n = 3) (**b**). Quantification of cerebral infarction volume (n = 3) (**c**). Quantification of brain swelling volume (n = 3) (**d**). Quantification of brain water content (n = 3) (**e**). Evaluation of neural injury in mice by using Zea Longa Neuroethology score (n = 12) (**f**). Scare bar: 1 cm. ^**^P < 0.01: ^***^P < 0.001νs. Sham: ^*#*^*P* < 0.05: ^*##*^*P* < 0.01: ^*###*^*P* < 0.001νs. Vehicle: ^*P* < 0.05 νs. NBP.

To investigate whether Nef exhibits BBB protective effects: tMCAO mouse as an in vivo model was established (Fig. 2a). Vehicle or Nef (30 mg/kg or 60 mg/kg) were administered to mice at 2 h and 12 h after restored the blood flow. TTC staining was employed to determine the brain infarction (Fig. 2b). The infarct volume: cerebral edema volume: and cerebral water content were increased in tMCAO mice: and Nef treatment exhibited a concentration-dependent decrease (Fig. 2c–e). Neurological status of mice was assessed using the Zea Longa neurological scoring system: Nef decreased the higher scores in tMCAO mice (Fig. 2f).

**Figure 3:**
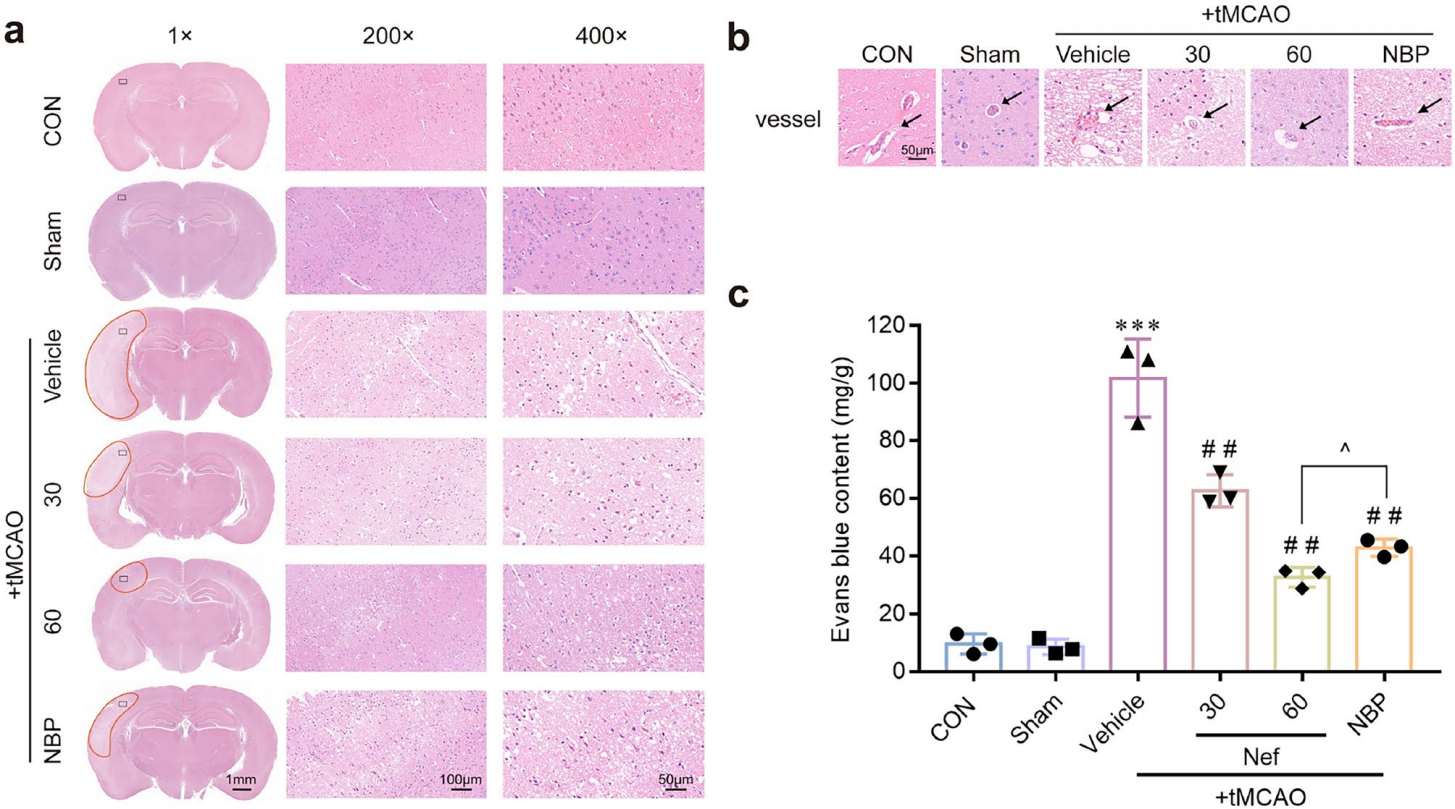
Effects of Nef on brain morphology of tMCAO mice. Examination of H&E stained sections provided insight into the morphological characteristics of mouse brains and cerebral vessels following tMCAO. Normal tissues were characterized by a purple-red coloration with rounded: full nuclei and a compact structure: while damaged tissue appeared white with pyknotic nuclei and the presence of numerous lacunae (n = 3) (**a**). Representative images of cerebral vessels (n = 3) (**b**). Quantification of brain Evans blue content (n = 3) (**c**). Scare bar: 50 μm. ^***^P < 0.001νs. Sham: ^*##*^*P* < 0.01νs. Vehicle: ^*P* < 0.05 νs. NBP (n = 3).

According to the results of H&E: tMCAO induced brain infarction: the peripheral neuron was nuclear pyknosis accompanied by the deeper staining: the penumbra area was swollen: neuropil vacuolation: glial cell hyperplasia: and Nef administration alleviated all the adverse phenomena elicited by tMCAO (Fig. 3a): dose-dependently. We also observed cerebral vessel damage in tMCAO mice (Fig. 3b): vessel walls integrity were impaired and the edges of the vessel wall were unclear. The mice administer Nef could effectively reverse the vessel injury. Brain EB content was quantified to further assess the integrity of BBB: tMCAO mice exhibited a marked increase in EB leakage: which was effectively attenuated by administration of Nef (Fig. 3c).

**Figure 4:**
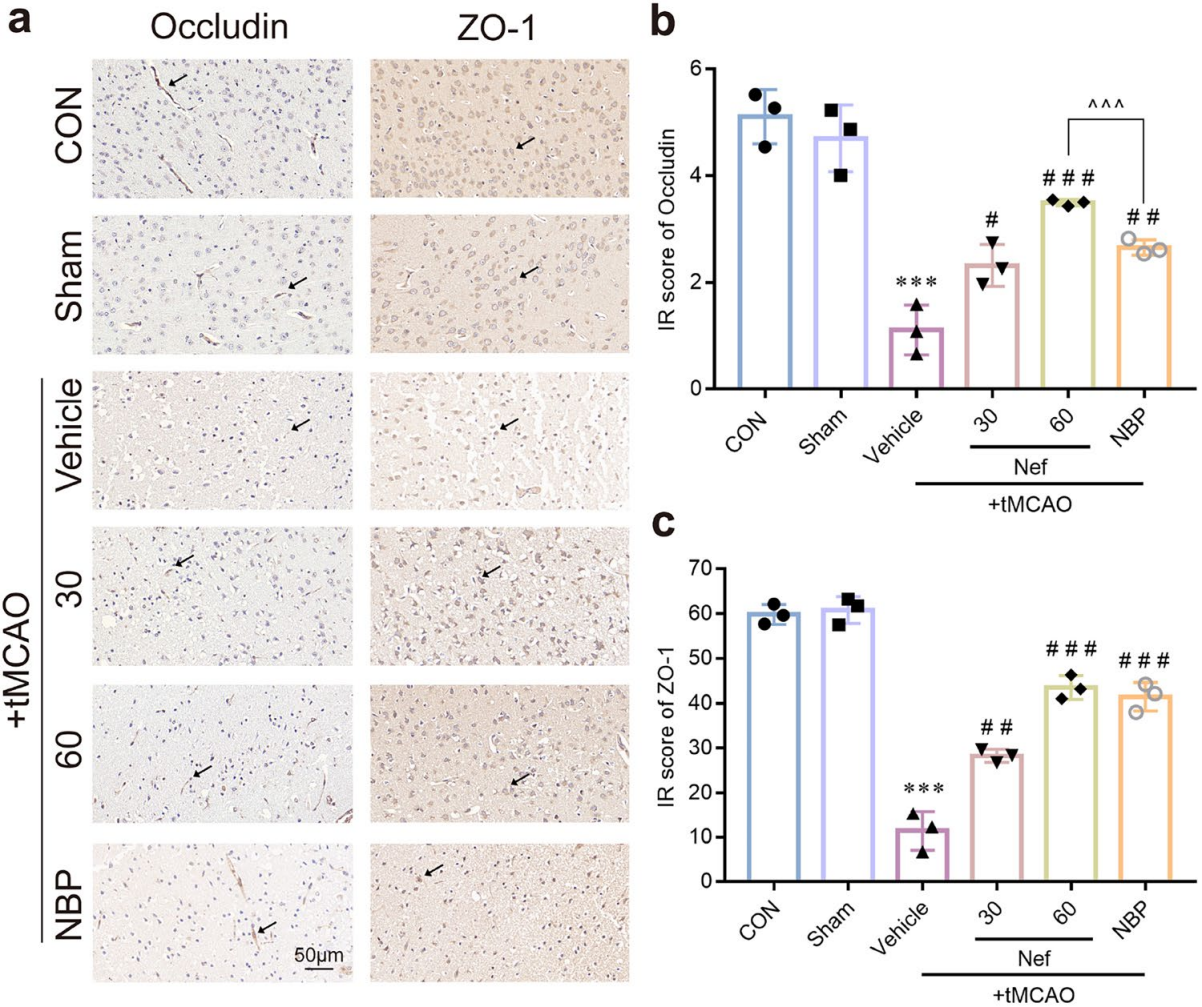
Effects of Nef on TJ proteins expression in mice underwent tMCAO. Representative micrographs of IHC staining (**a**). Quantitative analysis of the immunohistochemistry (**b**,**c**). Scare bar: 50 μm. ^***^P < 0.001νs. Sham: ^*#*^*P* < 0.05: ^*##*^*P* < 0.01: ^*###*^*P* < 0.001νs. Vehicle: ^^^*P* < 0.001 between treatment groups: (n = 3).

Immunohistochemistry demonstrated a decline in ZO-1 and Occludin expression levels post tMCAO (Fig. 4a–c).

**Figure 5:**
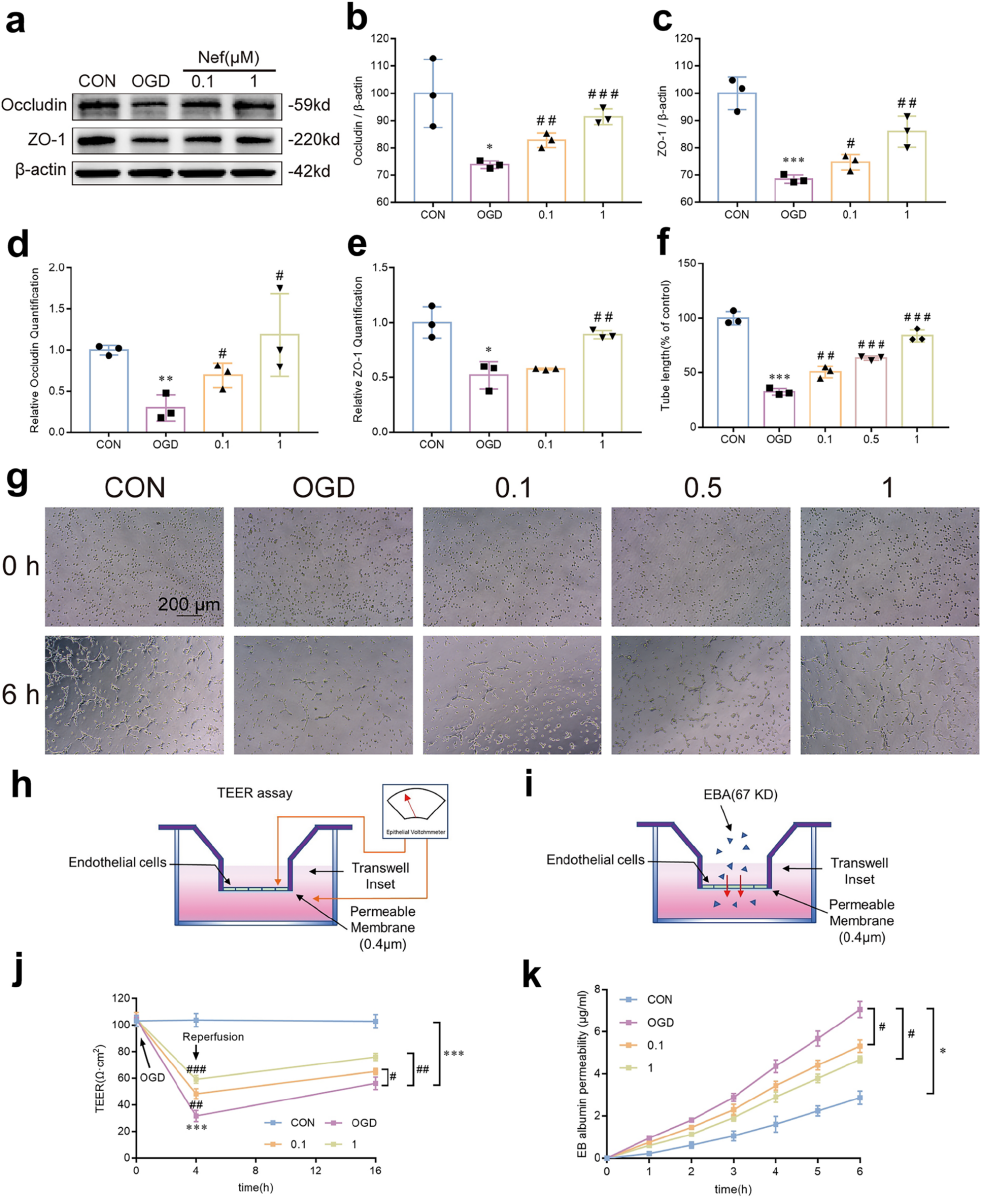
Effects of Nef on tube formation and endothelial barrier integrity under OGD/R condition. Representative Western blot bands of Occludin and ZO-1 (**a**). Quantitative analysis of the expressions of proteins(**b**,**c**). Relative mRNA expression quantification analysis (**d**,**e**). Quantitative analysis of tube branch length (**f**). Representative pictures depicting the formation of tubular structure (**g**). The TEER assay was employed to evaluate the barrier permeability of the bEnd.3 monolayer (**h**: **j**). EBA flux analysis was employed to evaluate the barrier permeability of the bEnd.3 monolayer (**i**: **k**). Scale bar: 200 μm. The experiments were performed in triplicate and repeated at least three times in different days. ^*^P < 0.05: ^**^P < 0.01: ^***^P < 0.001νs. Con: ^*#*^*P* < 0.05: ^*##*^*P* < 0.01: ^*###*^*P* < 0.001νs. OGD.

The gene and protein expression of ZO-1 and Occludin in bEnd.3 cells exposed to OGD/R. Consistently: Nef treatment reversed the diminished gene and protein expression of ZO-1 and Occludin in bEnd.3 cells (Fig. 5a–e)). The total pipe tube branch length of tube-like structures in bEnd.3 cells was measured to examine the tube formation ability. The morphology of the cells exhibited notable differences on tube-like structure and analysis data indicated the tubular structures decline in the total length after OGD/R. However: the tube branch length in cells which treated with Nef were dose-dependently increased (Fig. 5f,g). The protective effect of Nef on BBB was further assessed by TEER measurement (Fig. 5h) and EBA assay (Fig. 5i). Endothelial barrier permeability was evaluated after exposed to 4 h of OGD. Nef obviously ameliorated the reduction in TEER values at the period of reoxygenation onset and 12 h of reoxygenation compared with OGD group (Fig. 5j). A progressive increase in EBA extravasation is evident after OGD/R: and Nef treatment reversed this effect (Fig. 5k). These data confirm that Nef not only alleviated I/R induced brain injury but also protect cerebral vessel to improves BBB integrity after ischemic stroke.

### Nef activated PGC-1α to reduce mitochondrial oxidative stress and promote TJ proteins expression

**Figure 6:**
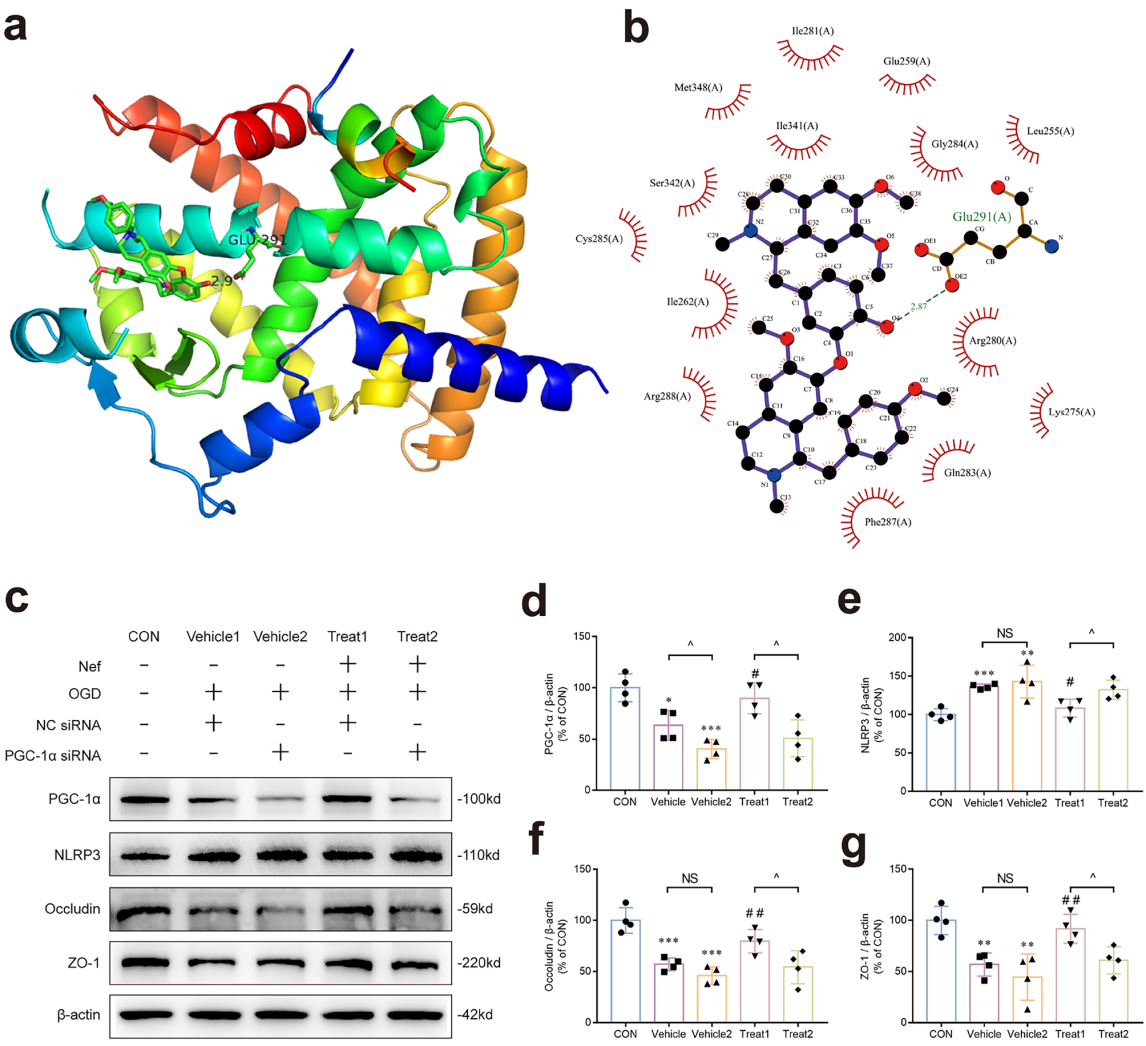
The protective effects of Nef in bEnd.3 cells is involved in promoting PGC-1α expression. Prediction of the 3D binding site and interactions was visualized using Pymol(**a**). Prediction of the 2D binding site and interactions was visualized using LigPlot + . Hydrogen bonds are indicated by lines colored in cyan. (**b**). PGC-1α knockdown significantly reduced Nef's inhibitory effect on NLRP3 protein and reduced the expression of TJ proteins elevated by Nef (**c**). Quantitative analysis of the relative proteins(**d**–**g**). ^*^P < 0.05: ^**^P < 0.01: ^***^P < 0.001 νs. CON: ^*#*^*P* < 0.05: ^*##*^*P* < 0.01 νs. Vehicle1: ^*P* < 0.05 between NC or PGC-1α siRNA transfection groups: (n = 4).

Molecular docking method was used to find the target protein interacting with Nef. To gain a comprehensive understanding with the docking residue sites of Nef and PGC-1α protein: Ligplots + and PyMOL softwares were utilized to generate visual representations of the corresponding protein residue in a 2D and 3D binding site (Fig. 6a,b). The conformation with the lowest energy was selected as the optimal conformation by the dock binding free energy. The docking simulation shows that Nef forms a hydrogen bonded with GLU-291: the best negative free energy of binding (− 9.1 kcal/mol) suggesting a possible interaction between Nef and PGC-1α (Table 1). In order to corroborate the findings of molecular docking: transient transfection of bEnd.3 cells with PGC-1α siRNA was conducted: followed by evaluation of the expression of PGC-1α: NLRP3: Occludin: and ZO-1 proteins (Fig. 6c). The results indicated that the inhibitory effect of Nef on NLRP3 expression and promotion of TJ proteins expression were counteracted by genetic PGC-1α knockdown (Fig. 6d–g)).

**Table 1:** Dock binding free energies (△Gb) and bonds of the docked compounds against proteins.

Compound	PDB ID	△Gb(kcal/mol)	Bonds formed between functional groups of component and protein residues		
			Functional groups	Protein residues	Bond
Neferine	3B1M	− 9.1	O	Glu291(A)	H-bond

**Figure 7:**
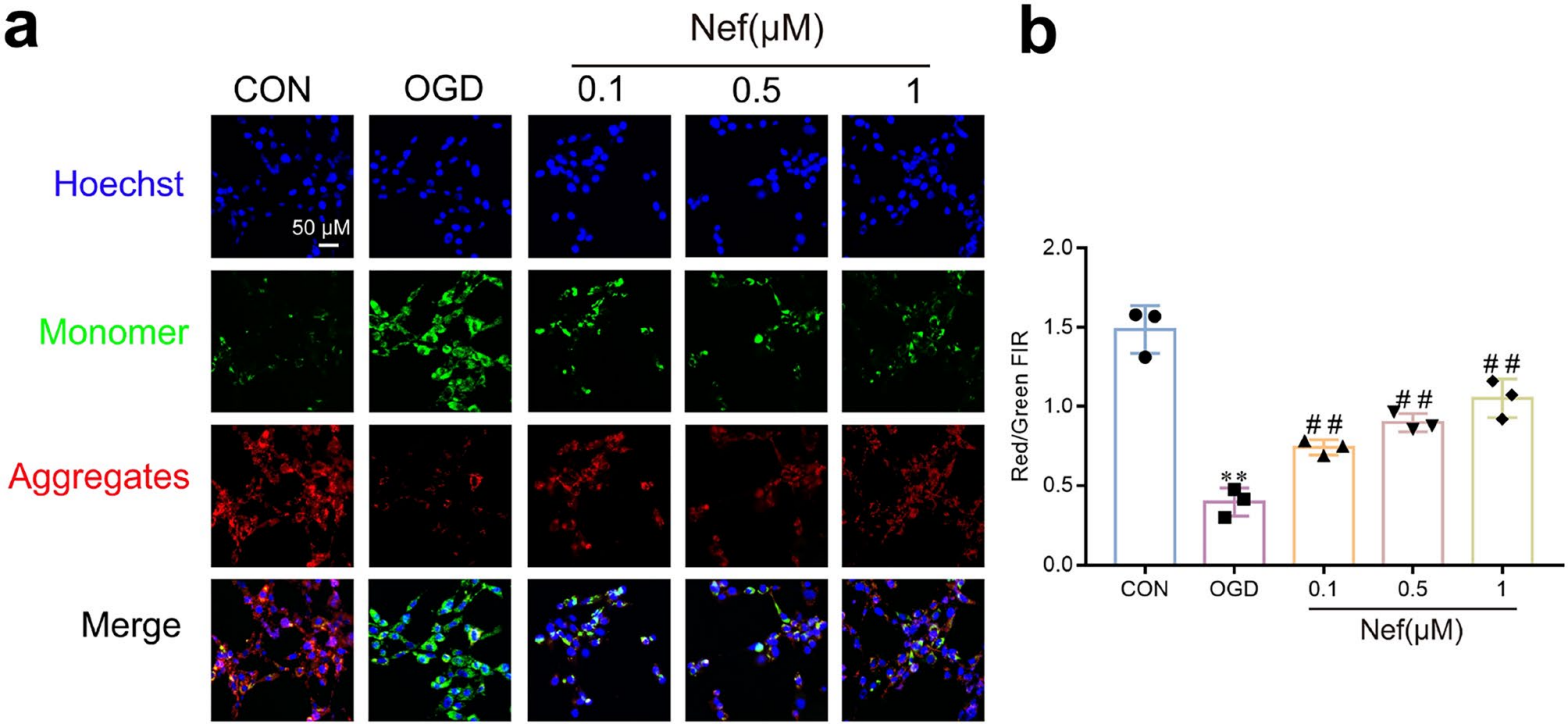
Effects of Nef on MMP in bEnd. 3 cells under OGD/R condition. Representative immunofluorescence staining of MMP in bEnd.3 cells (**a**). MMP as estimated by JC-1 red/green fluorescence intensity rate (FIR) (**b**). Scare bar: 50 μm. The experiments were performed in triplicate and repeated at least three times in different days. ^**^P < 0.01 νs. CON: ^*##*^*P* < 0.01 νs. OGD.

**Figure 8:**
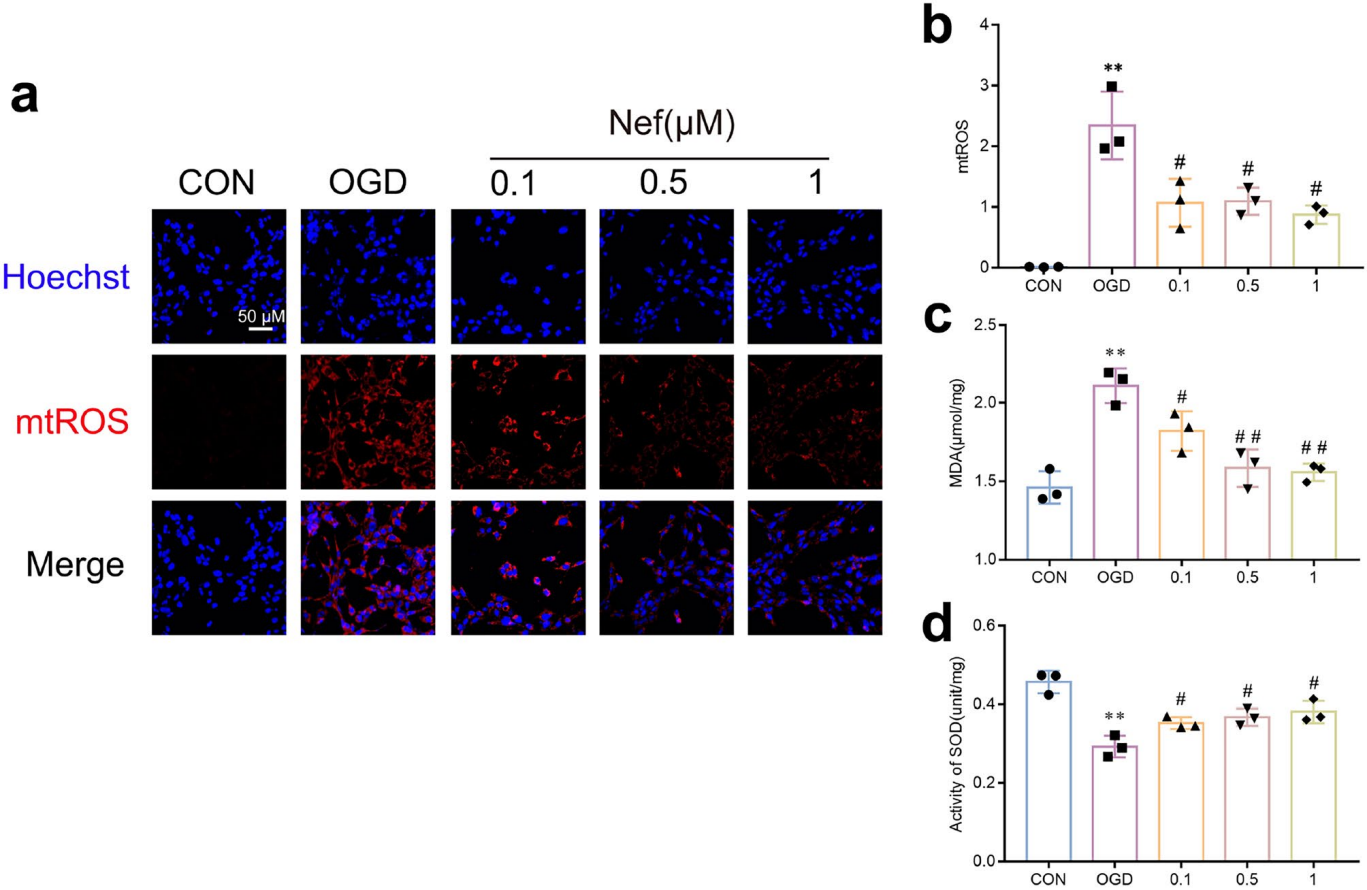
Effects of Nef on mtROS and intracellular oxidative stress in bEnd. 3 cells under OGD/R condition. Representative immunofluorescence of mtROS in bEnd.3 cells (**a**). Quantification of mtROS levels(**b**). Quantification of MDA and SOD represents cellular oxidative stress level (**c**,**d**). Scare bar = 50 μm. The experiments were performed in triplicate and repeated at least three times in different days. ^**^P < 0.01 νs. CON: ^*#*^*P* < 0.05: ^*##*^*P* < 0.01 νs. OGD.

PGC-1α is widely acknowledged as a predominant regulator of mitochondrial biogenesis and function: we evaluated mitochondrial function in bEnd.3 cells. Following OGD/R stimulation: MMP levels decreased and treated with Nef effectively reversed (Fig. 7a,b). We further assess the impact of Nef on mtROS: SOD and MDA in bend.3 cells. Notably: Nef treatment dose-dependently inhibited mtROS (Fig. 8a,b): promoted SOD (Fig. 8c) and MDA levels (Fig. 8d). These findings provide additional evidence supporting the role of Nef in reducing mtROS: improving oxidative stress damage: and enhancing mitochondrial function.

### Neferine inhibits hypoxic-induced BMECs pyroptosis via regulation of PGC-1α/NLRP3/GSDMD signaling pathway to maintain the integrity of BBB

**Figure 9:**
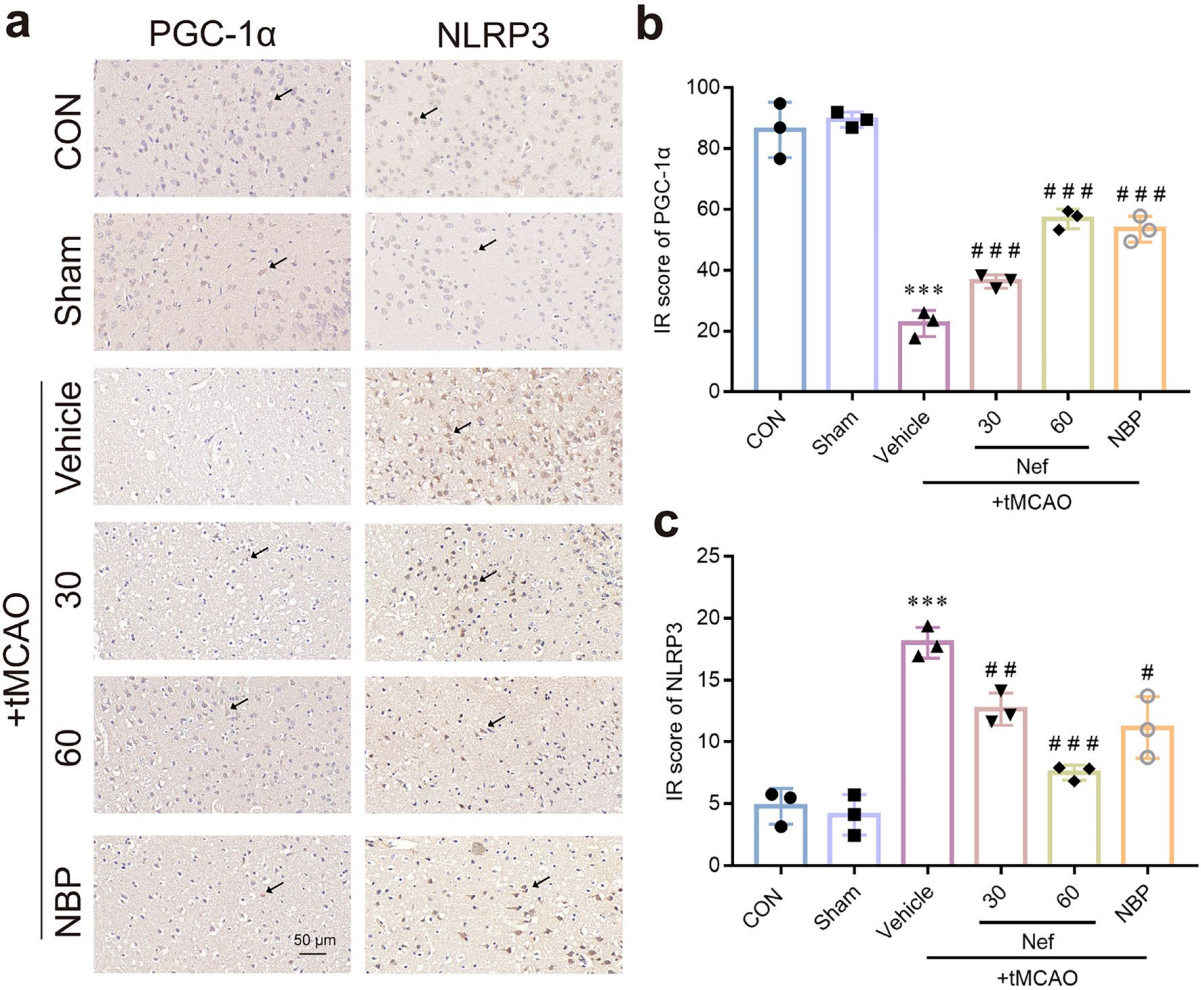
Effects of Nef on PGC-1α and NLRP3 proteins expression in mice underwent tMCAO. Representative micrographs of IHC staining (**a**). Quantitative analysis of the immunohistochemistry (**b**,**c**). Scare bar: 50 μm. ^***^P < 0.001νs. Sham: ^*#*^*P* < 0.05: ^*##*^*P* < 0.01: ^*###*^*P* < 0.001νs. Vehicle: ^^^*P* < 0.001 between treatment groups: (n = 3).

Immunohistochemistry was employed to analyze the expression of PGC-1α and NLRP3 in tMCAO mice. The results show that PGC-1α expression decreased and NLRP3 expression increased in I/R mice cerebral infarction region (Fig. 9a) and Nef dramatically reversed PGC-1α and NLRP3 expression (Fig. 9b,c).

**Figure 10:**
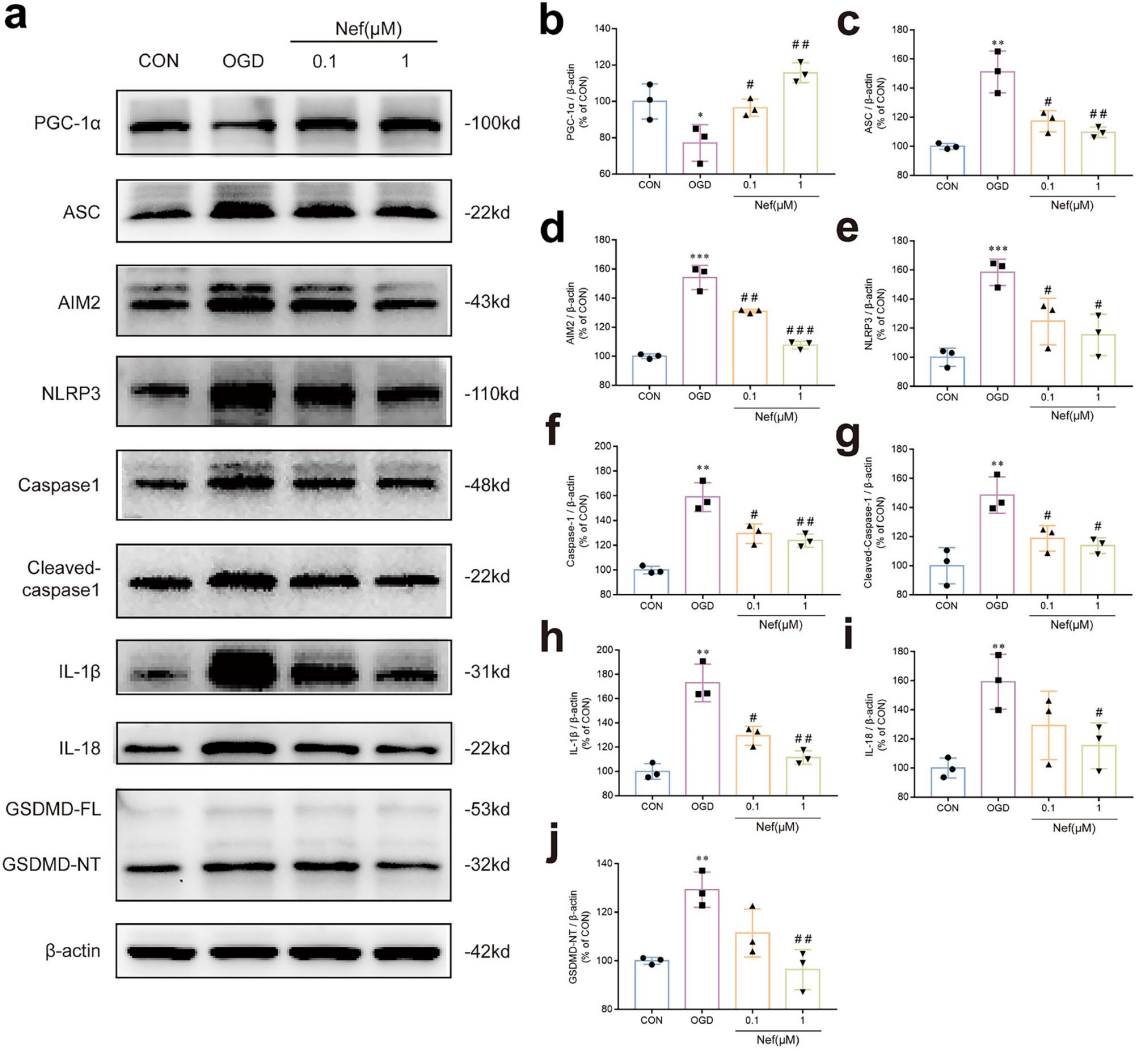
The regulatory effects of Nef on PGC-1α/NLRP3/GSDMD signaling pathway. Expression of PGC-1α and NLRP3 signaling pathway associated proteins (ASC: AIM2: NLRP3: Caspase-1: Cleaved caspase-1: IL-1β: IL-18: GSDMD) in bEnd.3 cells (**a**–**j**). ^*^P < 0.05: ^**^P < 0.01: ^***^P < 0.001νs. Con: ^*#*^*P* < 0.05: ^*##*^*P* < 0.01: ^*###*^*P* < 0.001νs. OGD: (n = 3).

To further verified the specific signaling pathway of Nef maintain the integrity of BBB: we also detected pyroptosis related proteins expression in bEnd.3 cells after OGD/R. The proteins expression of ASC: AIM2: Caspase-1: cleaved caspase-1: NLRP3: IL-1β: IL-18 and GSDMD were increased in bEnd.3 cells exposed to OGD/R and were reversed after the treated with Nef in vitro (Fig. 10). Consistent with the in vivo results: PGC-1α expression decreased in bEnd.3 cells under OGD/R and the expression was obviously upregulated by Nef. And the inhibiting effect of Nef on NLRP3 expression were cancelled by genetic PGC-1α knockdown (Fig. 6c). Together: these results demonstrated that Nef maintain the integrity of BBB by regulating PGC1-α/NLRP3/GSDMD mediated pyroptotic pathway.

## Discussion

**Figure 11:**
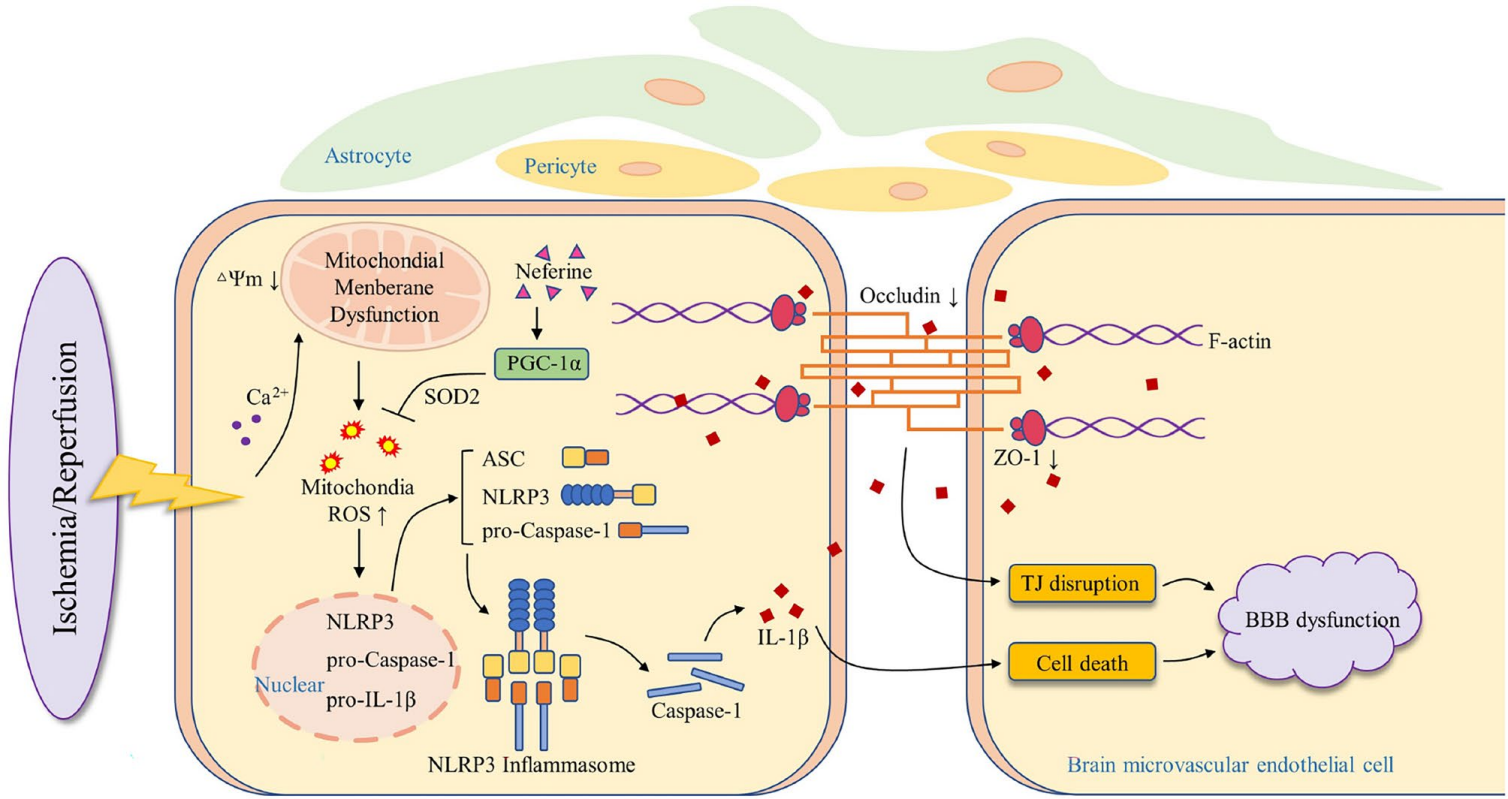
A schematic diagram is presented to illustrate the proposed mechanisms underlying the beneficial effects of Neferine in BMECs and its anti-ischemic activities. Nef activates PGC-1α: increases intracellular SOD activity and reduces the aggradation of mtROS: thereby inhibiting the assembly of the NLRP3 inflammasome and suppressing the cleavage and release of IL-1β and IL-18: further improving TJ disruption and BMECs pyroptosis. This effect of Nef leads to protect BMECs: BBB integrity and ischemic brain injury induced by ischemic stroke.

Previous researches have been shown that Nef had a powerful anti-inflammatory and neuroprotective effects^15–17^: so we tried to figure out the protective evidence of Nef on blood–brain barrier in cerebral I/R instead of neuroprotection. Our study using multiple methods to indicates that Nef repairs disruptive BBB to alleviated brain injury in cerebral I/R. Further investigation provided the first evidence that Nef target activate PGC-1α: and it promotes Occludin and ZO-1 expression: reduces NLRP3 inflammasome activation and mitochondrial oxidative stress induced pyroptosis to alleviates I/R injury of bEnd.3 cells: as illustrated in Fig. 11. Study also revealed that tight junction (TJ) protein mitigates attenuation and NLRP3 increased in bEnd.3 cells when PGC-1α was silenced. Taken all together: our work lends strong evidence supporting the protective effect of Nef on brain BMECs injury and maintain blood–brain barrier integrity after cerebral I/R.

Nef exhibits a beneficial effect on BMECs growth manifested in improving cell viability and tubular structure length and reducing the LDH release. EBA leakage and TEER values in vitro: cerebral vessel damage and EB leakage in vivo were observed to evaluate the effect of Nef on integrity of the BBB. The results both provided strong evidences of its positive regulation of BBB permeability. TJ proteins represented by Occludin and ZO-1 which are the mainly constituent and essential components in maintaining the fundamental function of the BBB. Our research shows that Nef increased TJ proteins expression which down regulated by I/R injury. This is consistent with the protective effect of Nef in tMCAO mice cerebral injury: it is manifested with the cerebral infarct volume mitigated: brain edema minimized: and neurobehavioral function enhanced.

Molecular docking results show us that Nef can bind to GLU-291 of PGC-1α (PDB ID: 3B1M) through hydrogen bonding: and the data suggested us PGC-1α may serve as the target protein mediating the protective effects of Nef on brain vascular endothelium after I/R. PGC-1α is highly expressed in BMECs^18^. In IS: PGC-1α was down-regulated: further reduced the capacity for mitochondrial oxidative phosphorylation and increased ROS production^4,5^. PGC-1α activation could promotes new angiogenesis: facilitating the delivery of oxygen and nutrients to ischemic tissues and preventing ROS overload^3^. In our research: mtROS production in bEnd.3 cells was increased and MMP values was decreased after OGD/R: all effects can be reversed by Nef. We also observed Nef could improve SOD expression and reduce MDA levels. Therefore: we infer that Nef may activate PGC-1α to inhibit the excessive aggregation of mtROS and maintain mitochondrial function.

Endogenous ROS levels may affect protein synthesis by regulating protein oxidative modification levels: so the increase of ROS results in the decrease of protein synthesis efficiency^19^. ROS promotes damage to Tight junction^20^. The upregulated of TJ proteins expression induced by Nef treatment may related to abolish the ROS overload.

ROS trigger the assembly of inflammasomes: leading to enhanced production and secretion of pro-inflammatory cytokines like IL-1β: exacerbating inflammatory response^20,21^. The activation of NLRP3 has been widely studied, it found to promote inflammatory cascade reactions: increase inflammatory factors release and aggravate vascular inflammation in IS^22,23^. The results also showed us that Nef suppressed NLRP3 inflammasome activation through activating PGC-1α and alleviated IS induced cerebral vascular endothelial injury. Up to now: it’s well known that GSDMD-N as a downstream molecule in pyroptosis: genetic PGC-1α knockdown to confirmed Nef interaction target and inhibit pyroptosis to protect blood–brain barrier. It is confirmed that Nef inhibited the expression of NLRP3 inflammasome and GSDMD in IS by targeting and activating of PGC-1α to maintain the integrity of BBB.

Based on the in vivo and in vitro research findings: we have concluded that Nef exerts a BBB-protective effect by rescue the impaired cerebral microvascular endothelial cells. The protective effect seems to be mediated by activating PGC-1α to reduce the excessive aggregation of mtROS and then inhibit the activation of NLRP3 inflammasome. Notably: the beneficial effects of Nef on cerebral microvascular endothelial against brain ischemia injury was first elucidated in our study: and first revealed that promoting PGC-1α expression could inhibit BMECs pyroptosis via PGC-1α/NLRP3/GSDMD pathway. In addition: we are concerned that PGC-1α has multiple functionalities beyond its role in reducing oxidative stress. Therefore: Further investigations are warranted to elucidate the impact of Nef on mitochondrial. To address these limitations and provide a stronger theoretical foundation for the application of Nef: we plan to conduct additional experiments and investigations. These endeavors will contribute to a deeper understanding on the mechanism of Nef’s anti-IS effect and offer more convincing evidence and theoretical backing.

## Materials and methods

### Ethics statement

This study utilized male C57BL/6 J mice procured from Changsheng Biotechnology Technology Co.: Ltd. (Liaoning: China). Standard environmental conditions were provided for the mice to minimize environmental variability. All methods were performed in accordance with the relevant guidelines and regulations: and approved by the Ethics Committee of Hubei University of Chinese Medicine (Approval No. HUCMS22702282).

### Establishment of the transient middle cerebral artery occlusion (tMCAO) model

A total of 90 male C57BL/6 J mice (aged 6–8 weeks and weighing 20–22 g) were used in short-term tMCAO experiments. Six groups were formed at random: control group: sham group: vehicle group: 30 mg/kg Nef group: 60 mg/kg Nef group: and 60 mg/kg NBP group.

Dl-3n-butylphthalide (NBP): a synthetic medication derived from *Apium graveolens Linn* seeds (celery): has been approved by the Food and Drug Administration (FDA) for phase II clinical trials in patients with acute ischemic stroke since 2016^24^. NBP has been found to possess diverse pharmacological actions: such as promoting cerebral microcirculation and improving mitochondrial function^25–27^. Due to its protective effect on BBB during IS^28^: it was adopted as a positive drug. Each group consisted of 12 animals that underwent a successful operation for subsequent experiments. A total of 1% of mice failed to achieve successful reperfusion following the tMCAO procedure: and approximately 20% died within one day.

The tMCAO procedure was carried out in accordance with the methods described previously^29^. To mitigate the influence of hormonal disturbances in female mice post-tMCAO surgery: only male mice were studied. Briefly: mice were anesthetized by intraperitoneal injection of pentobarbital sodium at a dose of 70 mg/kg: and a monofilament nylon suture with a round tip was inserted into the internal carotid artery via the right common carotid artery: avoid pterygopalatine artery and finally accessed the middle cerebral artery. It was placed for 45 min until the blood flow was restored by withdrawing the filament for reperfusion. The wounds were carefully sutured to prevent infection. The sham group underwent all surgical procedures: excluding the insertion of nylon sutures. Additionally: all mice were allowed free access to food and water.

### Drug administration

Neferine (Nef: CAS number 2292–16-2: purity over 98%) was obtained from Biopurify (Chengdu: China). The vehicle solution used for dissolving Nef consisted of 10% DMSO and 90% corn oil. Dl-3n-butylphthalide (NBP: CAS number 6066–49-5: purity over 95%) was obtained from Macklin (Shanghai: China). NBP was diluted with corn oil. At 2 and 12 h after surgery: separate groups of mice were treated with a volume of 0.2 mL via intragastric gavage of NEF (30: 60 mg/kg): NBP (60 mg/kg) or vehicle.

### Measurement of assessment

At the end of 24 h reperfusion: neurological status of mice was assessed using the Zea Longa neurological scoring system: as mentioned earlier^30^. For this study: mice with scores ranging from 1 to 3 were included as the tMCAO model mice.

Quantification of the infarct volume: cerebral edema volume: and cerebral water content.

At 24 h post-reperfusion: mice were euthanized by cervical dislocation. All brains were collected and divided into five sections: then immersed immediately in a 0.5% solution of 2,3,5-triphenyltetrazolium chloride (TTC: G1017: Servisebio: Wuhan: China) at 37 ℃ for approximately 30 min. A red stain appeared on normal brain tissues: while infarcted parts displayed in white. To correct the brain swelling volume due to cerebral edema: the area of the ipsilateral uninfarcted brain slice was subtracted from the contralateral hemisphere brain slice area to determine as infarct volume. Once the brain tissues were removed: any surplus water in the vicinity of the tissue was soaked up: and the weight was documented as wet weight. Afterward: a 72-h desiccation process in an oven set at 80 °C was employed to ensure complete removal of moisture in brain tissues. The weight obtained following the drying phase was denoted as the dry weight. Subtract dry weight from wet weight to determine brain water content.

### BBB permeability measurement

Mice were injected with 2% Evans Blue (E104208: Aladin: Shanghai: China) staining solution (4 ml/kg) through the tail vein 1 h before the end of reperfusion in accordance with the earlier descriptions to evaluate BBB permeability^31,32^: and blue coloration of the conjunctiva: ears: and limbs was observed. After complete anesthesia of mice: open the chest to completely expose the heart: excise the right atrial appendage: and saline solution was infused into the mice via the left ventricle: take the brain from the head after the fluid flowing out from the right atrium becomes clear. Cut out the brain tissue from the right ischemic area: add an appropriate amount of formamide in a ratio of 100: 1 for the brain tissue (mg): formamide (ml): and prepare the brain homogenate by thorough grinding using a homogenizer. The homogenate was incubated at 45 °C under light-protected conditions for 48 h. Afterward: the homogenate was centrifuged (1000 × g: 15 min) to obtain the supernatant. OD values at 632 nm were determined for each group utilizing a microplate reader (Tecan SunriseTM: Austria): and brain EB concentration of each group were measured by utilizing a standard curve as a reference.

### Histopathological examination

The whole brain tissue was fixed for 24 h in 4% paraformaldehyde at room temperature before undergoing paraffin embedding and subsequent sectioning into 5 µm thick coronal slices: including the cortical infarct region. Utilizing the Hematoxylin/Eosin (H&E) staining to provide a clear visualization of the tissue morphology and cellular architecture of the cortical region in the brain: and examine the stained sections of each group using an optical microscope (Olympus: Tokyo: Japan).

### Immunohistochemistry

Saline perfusion was carried out in the mice to eliminate blood from the vasculature: and then fix brain tissue with 4% paraformaldehyde. Following fixation: the tissues were dehydrated and sliced into 20 μm frozen sections. Sections were then blocked with 3% BSA (Vector Laboratories: Burlingame: CA: USA) for 30 min: and incubated with the following primary antibodies overnight at 4 °C: NLRP3: PGC-1α: Occludin: and ZO-1. After that: the sections were incubated with appropriate HPR-labeled secondary antibodies for 50 min at room temperature followed by with a 3 min staining of Hematoxylin dye solution (Servicebio: Wuhan: China) at room temperature. Fluorescence images of the specimen were acquired using the Olympus fluorescence microscope FV3000 and Image J software was employed for the measurement of fluorescence intensity. The working dilution of specific antibodies can be found in Supplementary materials.

### Cell cultures

Mouse brain endothelial cells (bEnd.3: No. CL-0598: Procell: Wuhan: China) were cultured in the complete endothelial cell medium (CM-0598: Procell: Wuhan: China) in a humidified incubator at 37 °C with 5% CO2 and 95% air.

### Oxygen–glucose deprivation/reoxygenation (OGD/R) establishment and drug treatment

Cells were subjected to OGD/R to simulate I/R injury. Upon reaching 80–90% confluence: bEnd.3 cells were placed in glucose-free DMEM (Procell: Wuhan: China) that had been degassed by ultrasonication. Then: cells were treated with Nef at concentrations of 0.1 μM: 0.5 μM or 1 μM: and transferred to a chamber consisting of 5% CO2 and 95% N2 for 9 h. Following the hypoxia exposure: replace the media with fresh growth medium and incubated another 12 h under normoxic conditions for reperfusion.

### Cell viability assays

A colorimetric CCK-8(GK10001: GLPBIO: CA: USA) assay was utilized for cell viability measurement. After OGD/R treatment: cell viability data exserts a relative percentage change compared to the untreated control group. Furthermore: a commercial assay kit (LDH: C0016: Beyotime: Shanghai: China) was employed to measure LDH release: a marker of cellular toxicity. Briefly: 50 µl of culture medium from each group was transferred to another 96-well plate: followed by LDH measurement using the manufacturer-provided test reagent. Additionally: the visualization of live and dead cells was conducted employing a Live/Dead assay kit (BB-4126: BestBio: Shanghai: China). Fluorescence images of the specimen were acquired using the Olympus fluorescence microscope FV3000 and Image J software was employed for the measurement of fluorescence intensity.

### Tube formation assay

The analysis of tube formation was conducted using Matrigel basement membrane matrix with growth factor-reduced (082,701: ABWbio: Shanghai: China). Briefly: a volume of 60 μL of Matrigel matrix was added to a 24-well plate. The pretreated bEnd.3 cells were then seeded and incubated for 6 h. Microscope photos were then obtained from three randomly chosen optical fields at a magnification of × 100. Analysis of the tube branch length was conducted by an uninformed experimenter: utilizing the “Angiogenesis Analyzer” plugin in Image J software. Each experiment was conducted a minimum of three times.

### Endothelial barrier permeability measurement

To assess endothelial barrier permeability: transmembrane electrical resistance (TEER) values of endothelial cell monolayer were measured using transmembrane resistance measuring instruments (EVOM2: WPI: Florida: USA) at time points of OGD: reoxygenation initiation: and 12 h of reperfusion. TEER values were calculated as followed:

TEER (Ω × cm^2^) = (total resistance − blank resistance) (Ω) × insert area (cm^2^).

The flux of Evans blue-labeled albumin (EBA: 1% BSA + 167.5 µg/ml Evans blue; 67 kDa) across the cell monolayer was measured according to previous report^33^. Following 12 h reoxygenation: 50 μL of EBA was added to the transwell inserts. Over the next six hours: media from the lower chambers from each group were collected hourly to measure optical density at 630 nm.

### Oxidative stress assay

Levels of superoxide dismutase (SOD: S0101S: Beyotime: Shanghai: China) and malondialdehyde (MDA: S0131S: Beyotime: Shanghai: China) were measured to assess intracellular oxidative stress using commercially available kits. The experiments were conducted in triplicate using 6-well plates for each condition.

### Western blot analysis

Cellular protein extraction was accomplished using Cell lysis buffer for Western and IP (P0013: Beyotime: Shanghai: China) in accordance with the manufacturer’s instructions. SDS-PAGE was employed to separate the protein samples (30 μg per group): followed by transferred onto a PVDF membrane (0.45 μm pore size: Millipore: MA: USA). The membrane blocked for 30 min and incubated overnight with the primary antibodies listed in Table 2 at 4 ℃. After that: the membrane were interacted with the respective secondary antibody for 1.5 h. β-actin was used as a loading reference. The protein bands were detected using ECL method (MA0186-1: Meilunbio: Dalian: China) and developed with gel densitometric scanning.

**Table 2:** The protein antibodies.

Antibody	Dilution ratio	Catalogue Number	Manufacturer
Occludin	1: 5000	66,378-1-Ig	Proteintech Group
ZO-1	1: 5000	21,773-1-AP	Proteintech Group
PGC-1α	1: 5000	66,369-1-Ig	Proteintech Group
NLRP3	1: 1000	15,101	CST
AIM2	1: 1000	63,660	CST
ASC/TMS1	1: 1000	67,824	CST
IL-1β	1: 1000	31,202	CST
Cleaved-Caspase-1	1: 1000	89,332	CST
Caspase-1	1: 1000	24,232	CST
GSDMD	1: 2000	bs-14287R	BIOSS
IL-18	1: 2000	bs-42148R	BIOSS
β-actin	1: 5000	bs-0061R	BIOSS

### Quantitative real-time polymerase chain reaction (qRT-PCR)

TRIzol reagent (BS258A: Biosharp: Anhui: China) was employed to obtain total RNA from bEnd.3 cells. Subsequently: DNA contamination was eliminated from the total RNA: and cDNA was synthesized using the ABScript II cDNA First Strand Synthesis Kit (RK20400: ABclone: Wuhan: China). In the end: cDNA was mixed with specific primers and qRT-PCR was performed using TOYOBO SYBR Green Realtime PCR Master Mix (QPK-201: TOYOBO: Japan) to validate the expression of specific mRNAs. The specific primers listed in Table 3 were utilized for PCR amplification of the cDNA fragment. The 2 − ΔΔCt method was used to calculate the relative gene expression levels: and β-actin expression served as endogenous internal control.

**Table 3:** The primer pairs.

Gene	Forward primer (5′ − 3′)	Reverse primer (5′ − 3′)
NLRP3	GCATTGCTTCGTAGATAGAGG	GAGAAGGACCCACAGTGTAA
PGC-1α	AGTTTTTGGTGAAATTGAGGAAT	TCATACTTGCTCTTGGTGGAAGC
Occludin	AGGACGGACCCTGACCACTA	CCTGCAGACCTGCATCAAAA
ZO-1	CACAAGGAGCCATTCCTGAAG	ATCACTAGGGGGCTCAGCAG
β-actin	CGTGCGTGACATCAAAGAGA	CCCAAGAAGGAAGGCTGGA

### Mitochondrial ROS (mtROS) measurement

The mtROS level was assessed with MitoSOX Red Mitochondrial Superoxide Indicator (M36008: Invitrogen: CA: USA). Following OGD/R injury: the cells underwent centrifugation at 1000 g for 5 min and were then washed with PBS. Mito-Tracker Red CMXRos and Hoechst 33,342 were then added and incubated in dark for 30 min. Fluorescence images of the specimen were acquired using the Olympus fluorescence microscope FV3000 and Image J software was employed for the measurement of fluorescence intensity.

### Mitochondrial membrane potential (MMP) measurement

Mitochondrial Membrane Potential Assay Kit (E-CK-A301: Elabscience: Wuhan: China) was employed to assess the MMP in bEnd.3 cell. Briefly: JC-1 dye solution was added to cells and incubated for 20 min after OGD/R injury: followed by 5 min incubation with Hoechst 33,342. Olympus Fluorescence FV3000 microscope was employed to observe the fluorescence and Image J software was employed for the measurement of fluorescence intensity.

### Molecular docking

To initiate the molecular docking process: the PGC-1α protein structure (PDBID: 3B1M) was imported from the RSCB PDB database in PDB format: while the 3D structure of Neferine was obtained from PubChem. Using PyMOL software: water molecules were removed: and the ligand was isolated. After employing AutoDock Tools 1.5.6 to perform tasks such as hydrogen addition: charge calculation: and atomic type assignment. Subsequently: AutoDock Vina 1.1.2 was utilized for molecular docking simulations to investigate the binding characteristics between the Nef ligand and PGC-1α protein. The binding energy: which serves as an evaluation index for molecular docking: was calculated. The optimal conformations of Nef and PGC-1α were determined by analyzing the clusters within the docking results: which were selected based on their respective binding energies. Typically: a lower docking energy indicates a stronger interaction force between the components: and a threshold of -7 kcal/mol is often used. The epitope with the lowest affinity scores predicted by AutoDock Vina was subjected to LigPlot + and PyMol for further visualization of the interactions.

### Short interfering (Si) RNA and plasmid transfection

Cells cultivated in a 6-well plate with OPTI medium (31,985,062: Invitrogen: CA: USA) were transfected with either PGC-1α siRNA (3′- CCG CAA UUC UCC CUU GUA UTT-5′) or negative control siRNA (3′-ACG UGA CAC GUU CGG AGA A-5′) using Lipofectamine 3000 transfection reagent (L3000001: Invitrogen: CA: USA). The siRNAs were obtained from Sangon (Shanghai: China). Following 12 h incubation: all medium was substituted with growth medium: and cells were cultured for an additional 24 h. Cells were then subjected to OGD/R injury and harvested for Western blot analysis.

### Statistical analysis

GraphPad Prism 7 Software (La Jolla: CA: USA) was used for statistical analysis: and all results were expressed as means ± SD. One-way ANOVA with Bonferroni’s test was utilized to determine the differences between groups for data with a single dosage or time point. For comparisons between two groups: including OGD and drug treatment: PGC-1α siRNA and drug treatment: the t-test was used. Statistical significance was defined as a *P*-value of 0.05 or less.

### Supplementary Information

Supplementary Information.

## Data Availability

The datasets used and analyzed during the current study are available in the science data bank (10.57760/sciencedb.14510).

## Abbreviations

IS: Ischemic stroke
BBB: Blood–brain barrier
CCA: Common carotid artery
CNS: Central nervous system
EB: Evans blue
ECA: External carotid artery
FBS: Fetal bovine serum
FDA: Food and drug administration
ICA: Internal carotid artery
LDH: Lactate dehydrogenase
MDA: Malondialdehyde
MMP: Mitochondrial membrane potential
NLRP3: NOD-like receptors family, pyrin domaincontaining 3
OGD/R: Oxygen and glucose-deprivation/reperfusion
PPA: Pterygopalatine artery
SOD: Superoxide dismutase

## References

[1] He Z, Ning N, Zhou Q, Khoshnam SE, Farzaneh M (2020). Mitochondria as a therapeutic target for ischemic stroke. Free Radic. Biol. Med..

[2] Jia J, Jin H, Nan D, Yu W, Huang Y (2021). New insights into targeting mitochondria in ischemic injury. Apoptosis.

[3] Abu SO, Arroum T, Morris S, Busch KB (2023). PGC-1α Is a master regulator of mitochondrial lifecycle and ROS stress response. Antioxidants.

[4] Xu Y (2018). The PGC-1α Activator ZLN005 Ameliorates Ischemia-Induced Neuronal Injury In Vitro and In Vivo. Cell. Mol. Neurobiol..

[5] Chen S-D (2010). Activation of calcium/calmodulin-dependent protein kinase IV and peroxisome proliferator-activated receptor γ coactivator-1α signaling pathway protects against neuronal injury and promotes mitochondrial biogenesis in the hippocampal CA1 subfield after transient global ischemia. J. Neurosci. Res..

[6] Bi J (2019). Irisin alleviates liver ischemia-reperfusion injury by inhibiting excessive mitochondrial fission: promoting mitochondrial biogenesis and decreasing oxidative stress. Redox Biol..

[7] Li Y (2021). Songorine promotes cardiac mitochondrial biogenesis via Nrf2 induction during sepsis. Redox Biol..

[8] Kang J (2018). Radiation-induced overexpression of transthyretin inhibits retinol-mediated hippocampal neurogenesis. Sci. Rep..

[9] Zhou Y-J (2013). Neferine exerts its antithrombotic effect by inhibiting platelet aggregation and promoting dissociation of platelet aggregates. Thromb. Res..

[10] Marthandam AS, Mariappan R, Muthusamy S, BK Velmurugan (2018). Pharmacological benefits of neferine - A comprehensive review. Life Sci..

[11] Wu C (2019). Mitochondrial protective effect of neferine through the modulation of nuclear factor erythroid 2-related factor 2 signalling in ischaemic stroke. Br. J. Pharmacol..

[12] Sengking J (2022). Protective Effect of Neferine in Permanent Cerebral Ischemic Rats via Anti-Oxidative and Anti-Apoptotic Mechanisms. Neurotox. Res..

[13] Guolan D (2018). Anti-inflammatory effects of neferine on LPS-induced human endothelium via MAPK: And NF-κβ pathways. Pharmazie.

[14] Tang Y-S (2019). Neferine inhibits LPS-ATP-induced endothelial cell pyroptosis via regulation of ROS/NLRP3/Caspase-1 signaling pathway. Inflamm. Res..

[15] Wang M-Y (2023). Neferine ameliorates nonalcoholic steatohepatitis through regulating AMPK pathway. Phytomedicine.

[16] Wu X-L (2020). Neferine alleviates memory and cognitive dysfunction in diabetic mice through modulation of the NLRP3 inflammasome pathway and alleviation of endoplasmic-reticulum stress. Int. Immunopharmacol..

[17] Lin T-Y (2022). Neferine: an Alkaloid from Lotus Seed Embryos: Exerts Antiseizure and neuroprotective effects in a Kainic acid-induced seizure model in rats. Int. J. Mol. Sci..

[18] Wang Y (2021). Medioresinol as a novel PGC-1α activator prevents pyroptosis of endothelial cells in ischemic stroke through PPARα-GOT1 axis. Pharmacol. Res..

[19] Topf U (2018). Quantitative proteomics identifies redox switches for global translation modulation by mitochondrially produced reactive oxygen species. Nat. Commun..

[20] Yokoo K, Yamamoto Y, Suzuki T (2021). Ammonia impairs tight junction barriers by inducing mitochondrial dysfunction in Caco-2 cells. FASEB J..

[21] Fu J, Wu H (2023). Structural Mechanisms of NLRP3 Inflammasome assembly and activation. Annu. Rev. Immunol..

[22] Poh L (2022). The role of inflammasomes in vascular cognitive impairment. Mol. Neurodegener..

[23] Wu AG (2021). Targeting microglial autophagic degradation in NLRP3 inflammasome-mediated neurodegenerative diseases. Ageing Res. Rev..

[24] Wang S (2018). Dl-3-n-Butylphthalide (NBP): A promising therapeutic agent for ischemic stroke. CNS Neurol. Disord. Drug Targets.

[25] Liu X (2021). Dl-3-n-butylphthalide inhibits neuroinflammation by stimulating foxp3 and Ki-67 in an ischemic stroke model. Aging (Albany NY).

[26] Huang L (2018). From stroke to neurodegenerative diseases: The multi-target neuroprotective effects of 3-n-butylphthalide and its derivatives. Pharmacol. Res..

[27] Wang A (2023). Efficacy and Safety of Butylphthalide in Patients With Acute Ischemic Stroke: A randomized clinical trial. JAMA Neurol..

[28] Mamtilahun M (2020). DL-3n-Butylphthalide improves blood-brain barrier integrity in rat after middle cerebral artery occlusion. Front. Cell Neurosci..

[29] Dai M-J (2023). Dl-3-n-butylphthalide promotes angiogenesis in ischemic stroke mice through upregulating autocrine and paracrine sonic hedgehog. Acta Pharmacol. Sin..

[30] Shen W (2021). Treadmill exercise enhances synaptic plasticity in the ischemic penumbra of MCAO mice by inducing the expression of Camk2a via CYFIP1 upregulation. Life Sci..

[31] Gong S (2021). Endothelial conditional knockdown of NMMHC IIA (Nonmuscle myosin heavy Chain IIA) attenuates blood-brain barrier damage during ischemia-reperfusion Injury. Stroke.

[32] Chen X-Y (2021). Inhibition of the immunoproteasome LMP2 ameliorates ischemia/hypoxia-induced blood-brain barrier injury through the Wnt/β-catenin signalling pathway. Mil. Med. Res..

[33] Barna L (2020). Simvastatin: edaravone and dexamethasone protect against kainate-induced brain endothelial cell damage. Fluids Barriers CNS.

