# Supplementary material for: Neferine inhibits BMECs pyroptosis and maintains blood–brain barrier integrity in ischemic stroke by triggering a cascade reaction of PGC-1α
Source: Sci Rep. 2024 Jun 23;14:14438. doi: 10.1038/s41598-024-64815-w (PMC11194274; doi:10.1038/s41598-024-64815-w)
Supplement: Supplementary file 1 — Supplementary Information. [file 41598_2024_64815_MOESM1_ESM.docx]

**Immunohistochemistry**

The working dilution of primary antibodys:

anti-NLRP3 Goat Polyclonal antibody (1:100, Servicebio, Wuhan, China), anti-PGC-1α Mouse Polyclonal antibody (1:100, Servicebio, Wuhan, China), anti-Occludin Mouse Polyclonal antibody (1:100, Servicebio, Wuhan, China), anti-ZO-1 Rabbit Polyclonal antibody (1:100, Servicebio, Wuhan, China)

The working dilution of secondary antibodys:

HRP-labeled rabbit anti-goat IgG (1:200, Servicebio, Wuhan, China) and either HRP-labeled goat anti-rabbit IgG (1:200, Servicebio, Wuhan, China) or HRP-labeled goat anti-mouse IgG (1:200, Servicebio, Wuhan, China)

**Supplementary figures:**


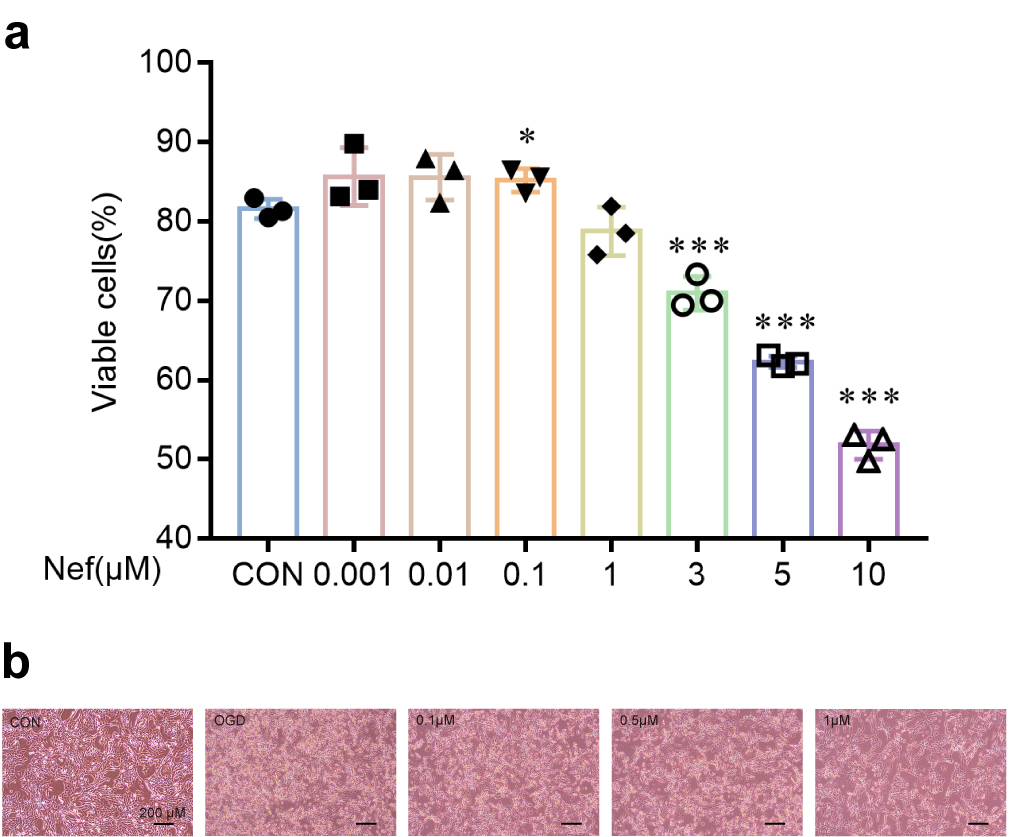


Figure S1：(A) The effect of Nef on the viability of bEnd.3 cells was revealed through the CCK8 assay, with a concentration range of 0.001μM to 10μM. (B) The cell viability was measured after 4–10 h of OGD by CCK8. (C) The morphology and growth status of cells in each group after 12 hours of reperfusion. ∗p < 0.05, ∗∗p < 0.01, ∗∗∗p < 0.001νs. Con, (n = 3).


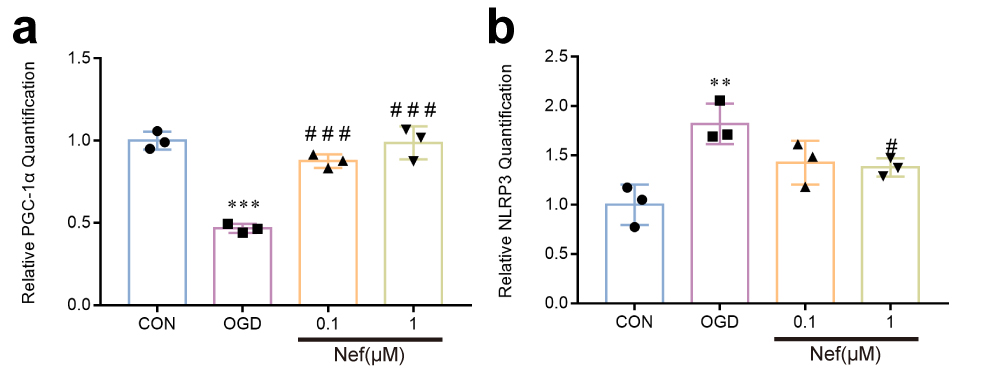


Figure S2：(A) The mRNA level of PGC-1α in each group. (B) The mRNA level of NLRP3 in each group. ∗p < 0.05, ∗∗p < 0.01, ∗∗∗p < 0.001νs. Con, #p < 0.05, ##p < 0.01, ###p < 0.001νs. OGD, (n = 3).

**Original western blots**


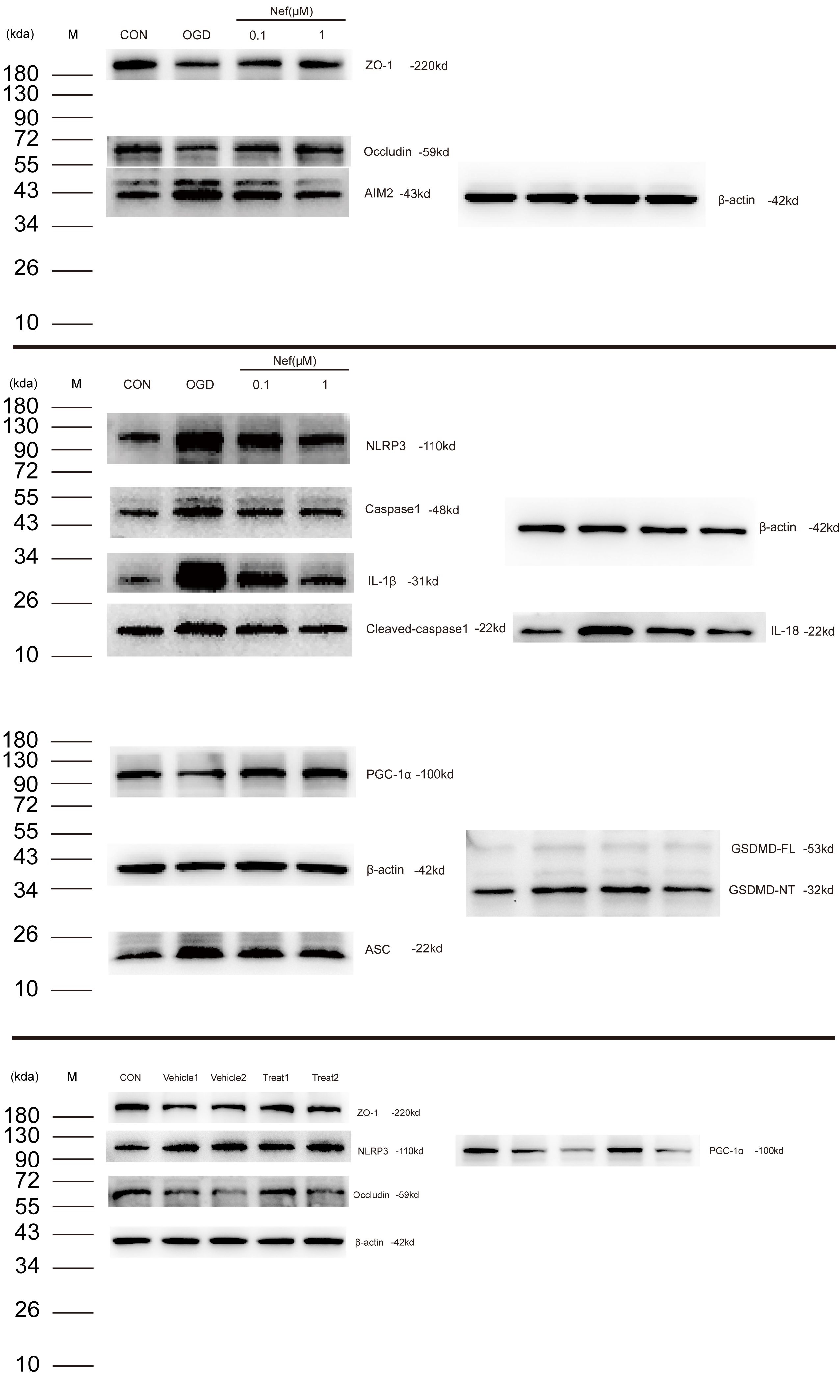


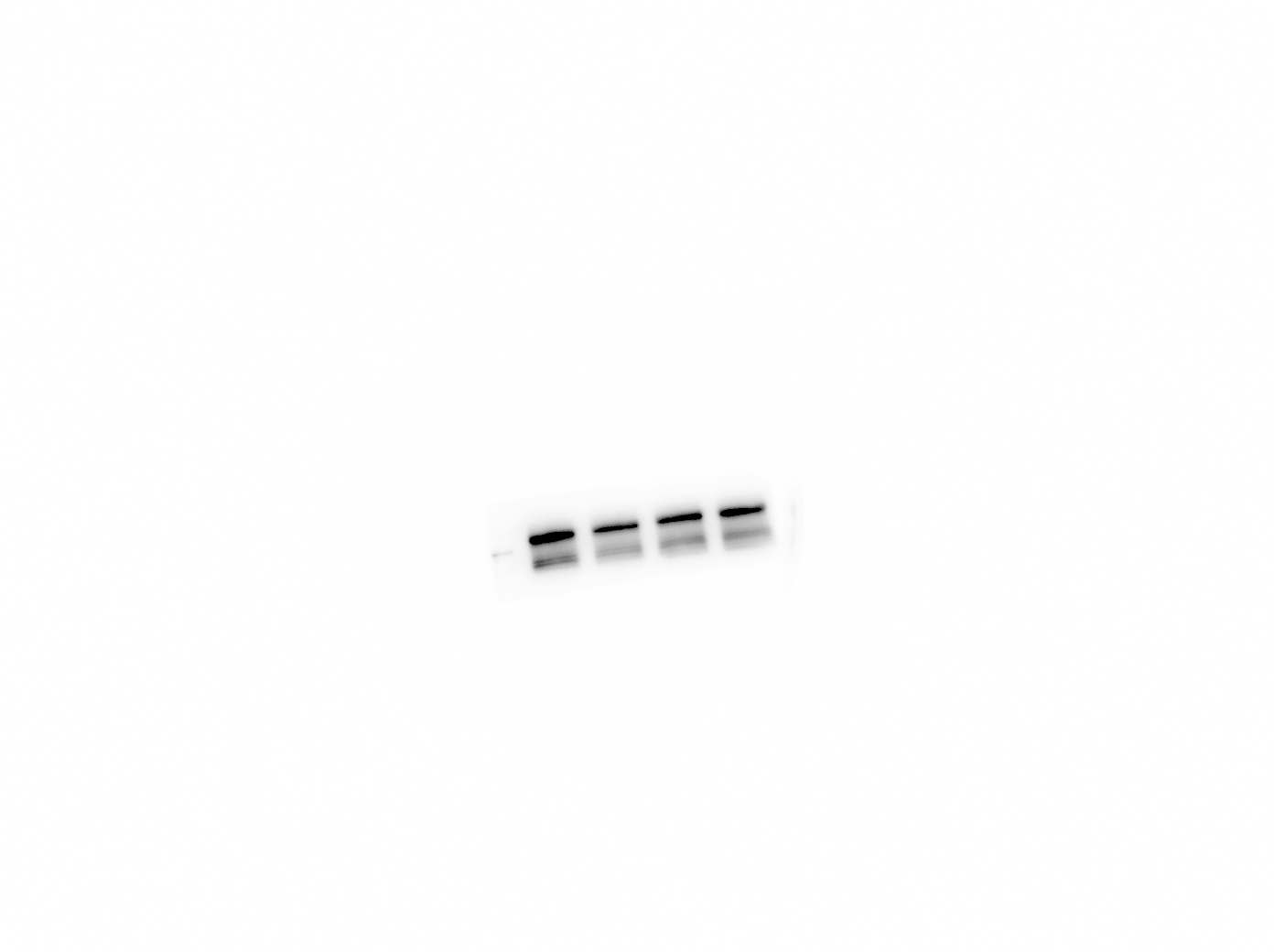


180

(kda) M CON OGD 0.1 1

Figure5-ZO-1，240kda


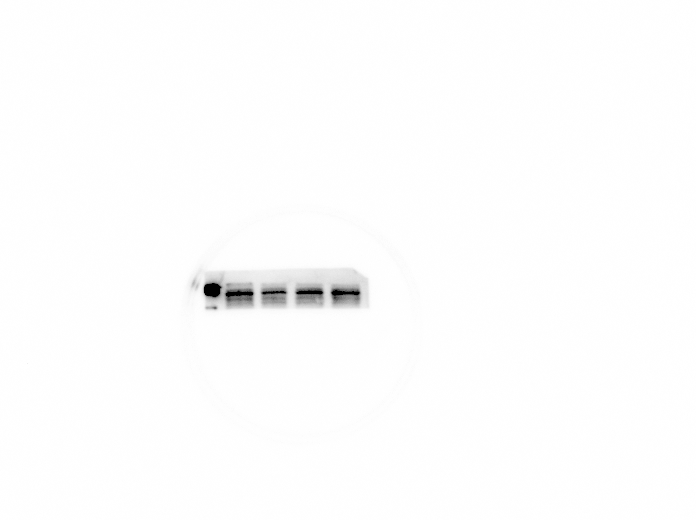


(kda) M CON OGD 0.1 1

55

72

72

(kda) M CON OGD 0.1 1

Figure5-Occludin 59kda


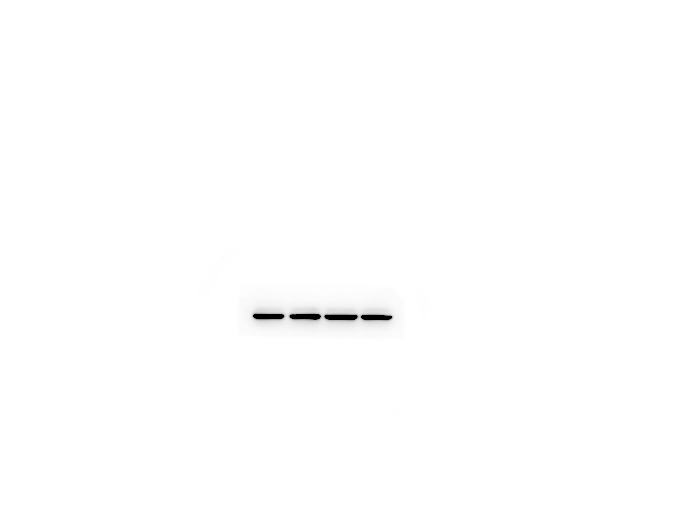


43

34

(kda) M CON OGD 0.1 1

Figure5-β-actin 42kda


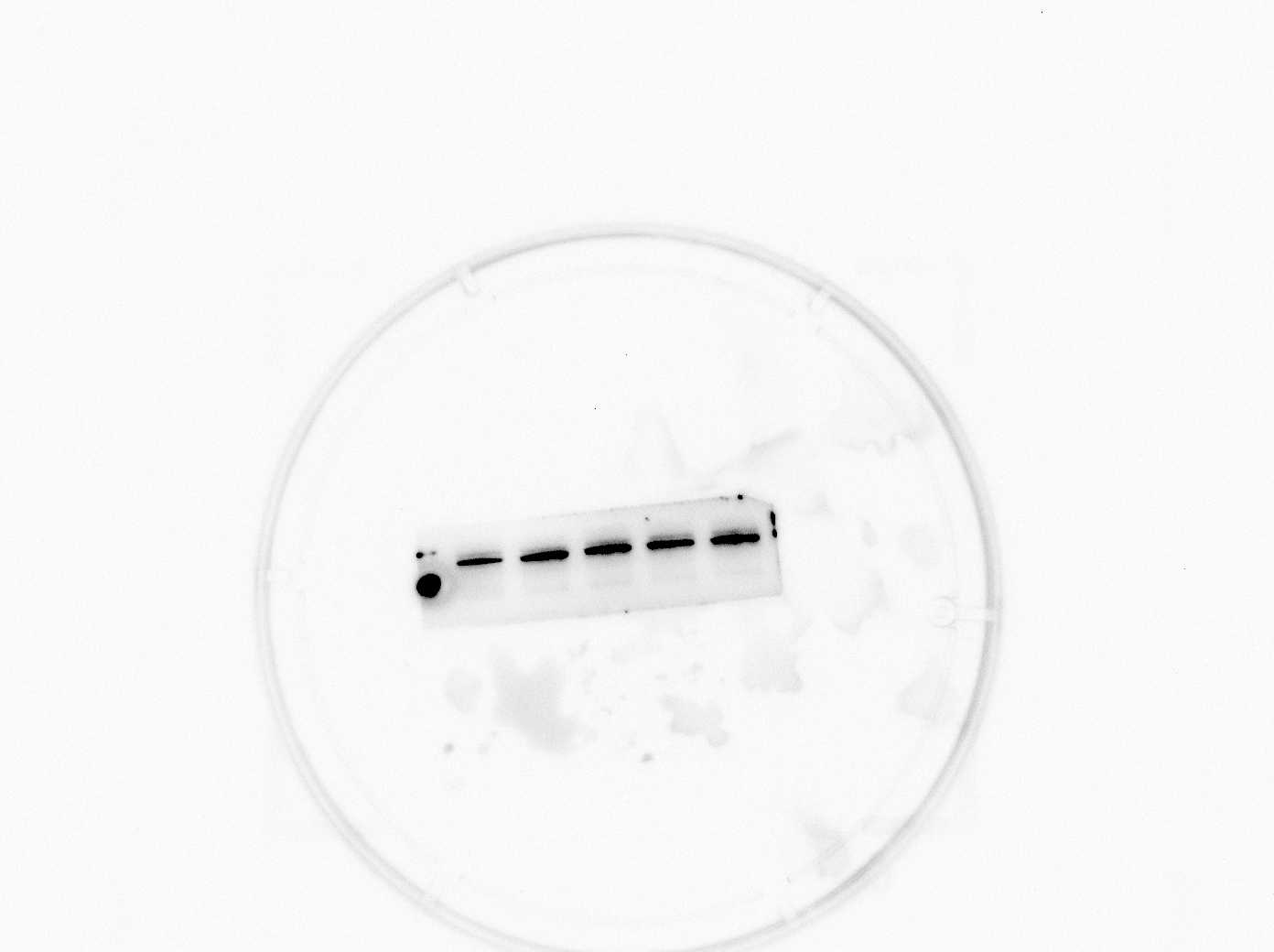


110

90

(kda) M CON Veh1 Veh2 Tre1 Tre2

Figure6-NLRP3 110kda


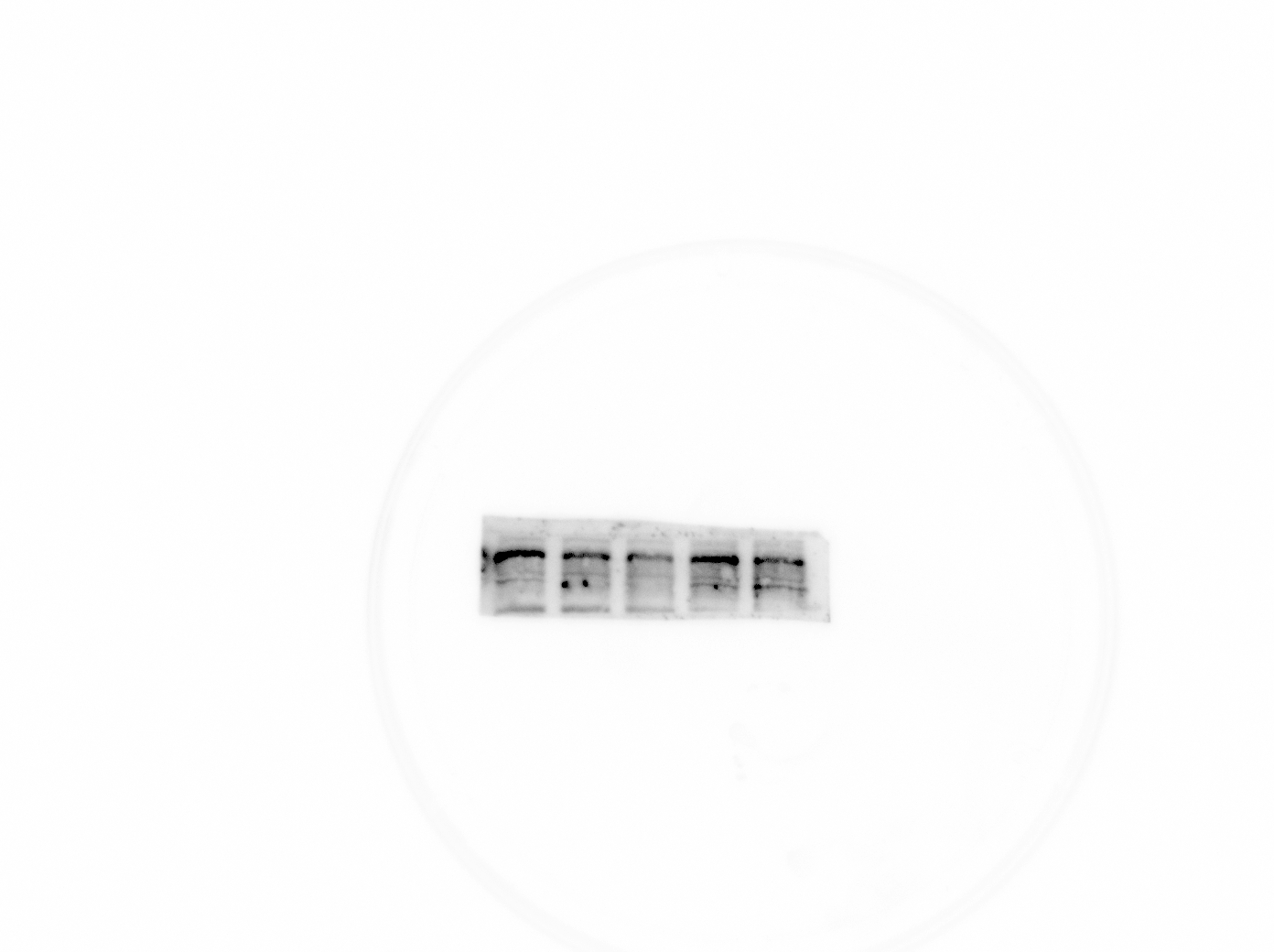


72

55

(kda) M CON Veh1 Veh2 Tre1 Tre2

Figure6-Occludin 59kda


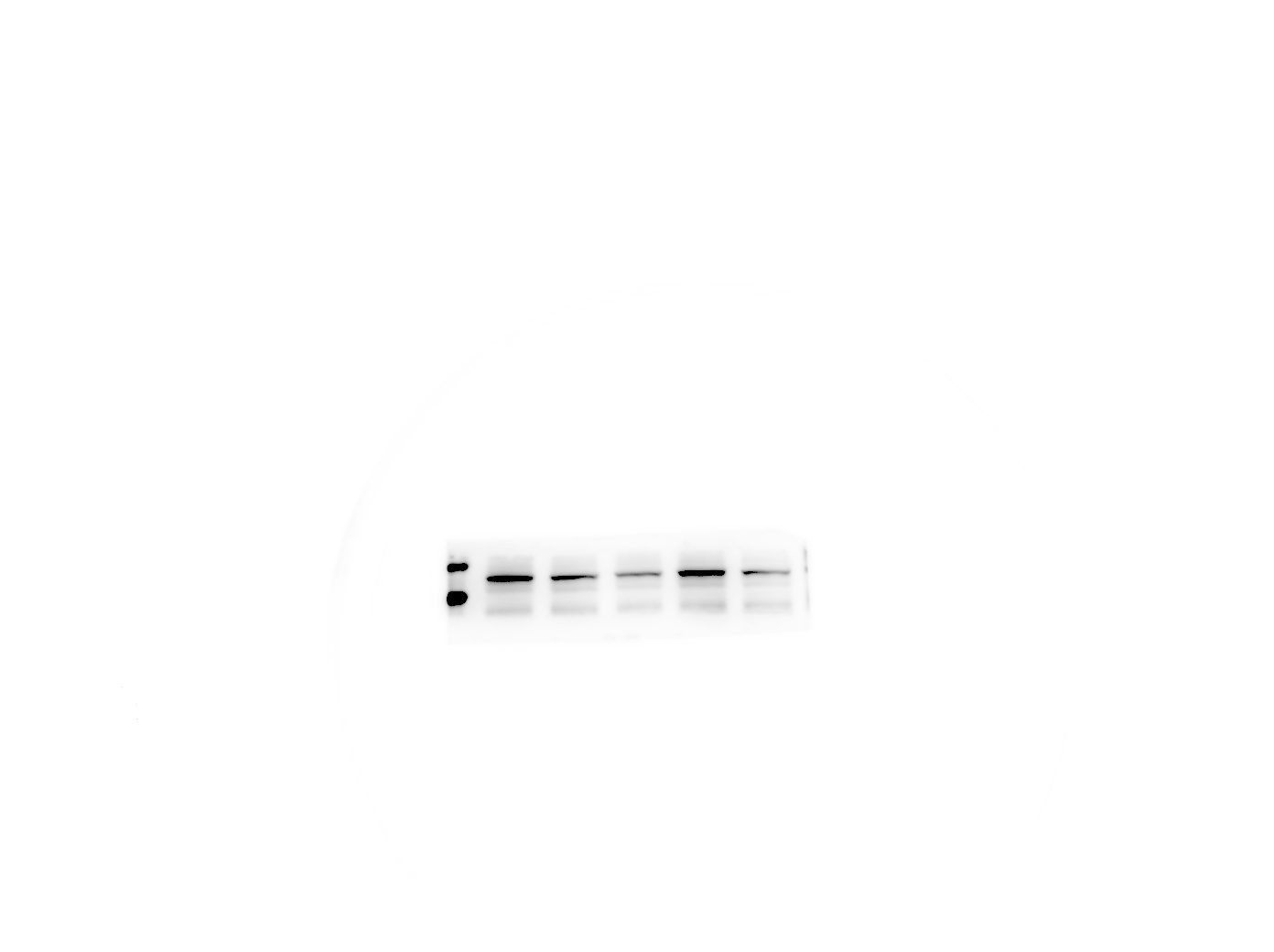


(kda) M CON Veh1 Veh2 Tre1 Tre2

90

130

Figure6-PGC-1α 100kda


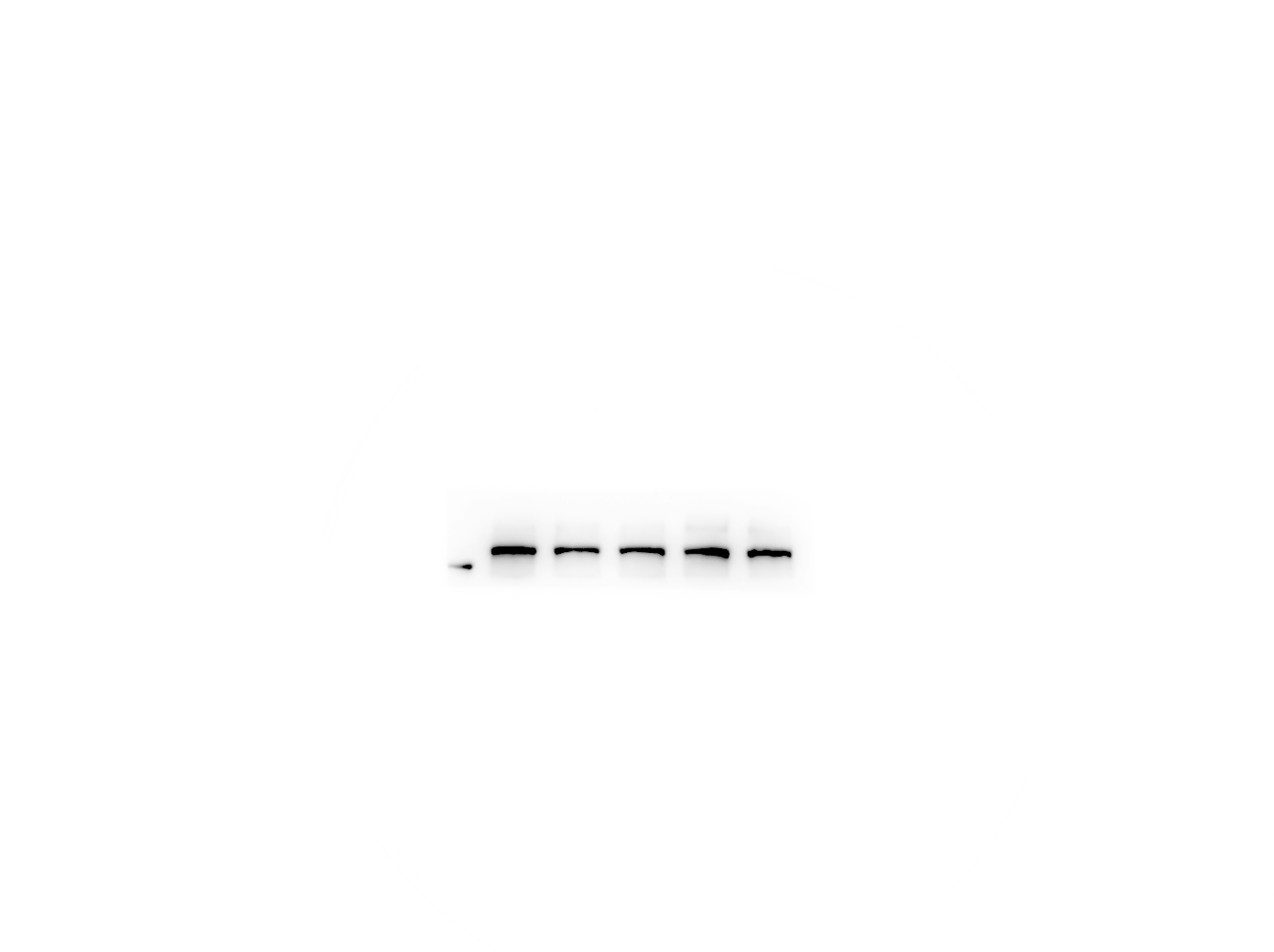


180

(kda) M CON Veh1 Veh2 Tre1 Tre2

Figure6-ZO-1


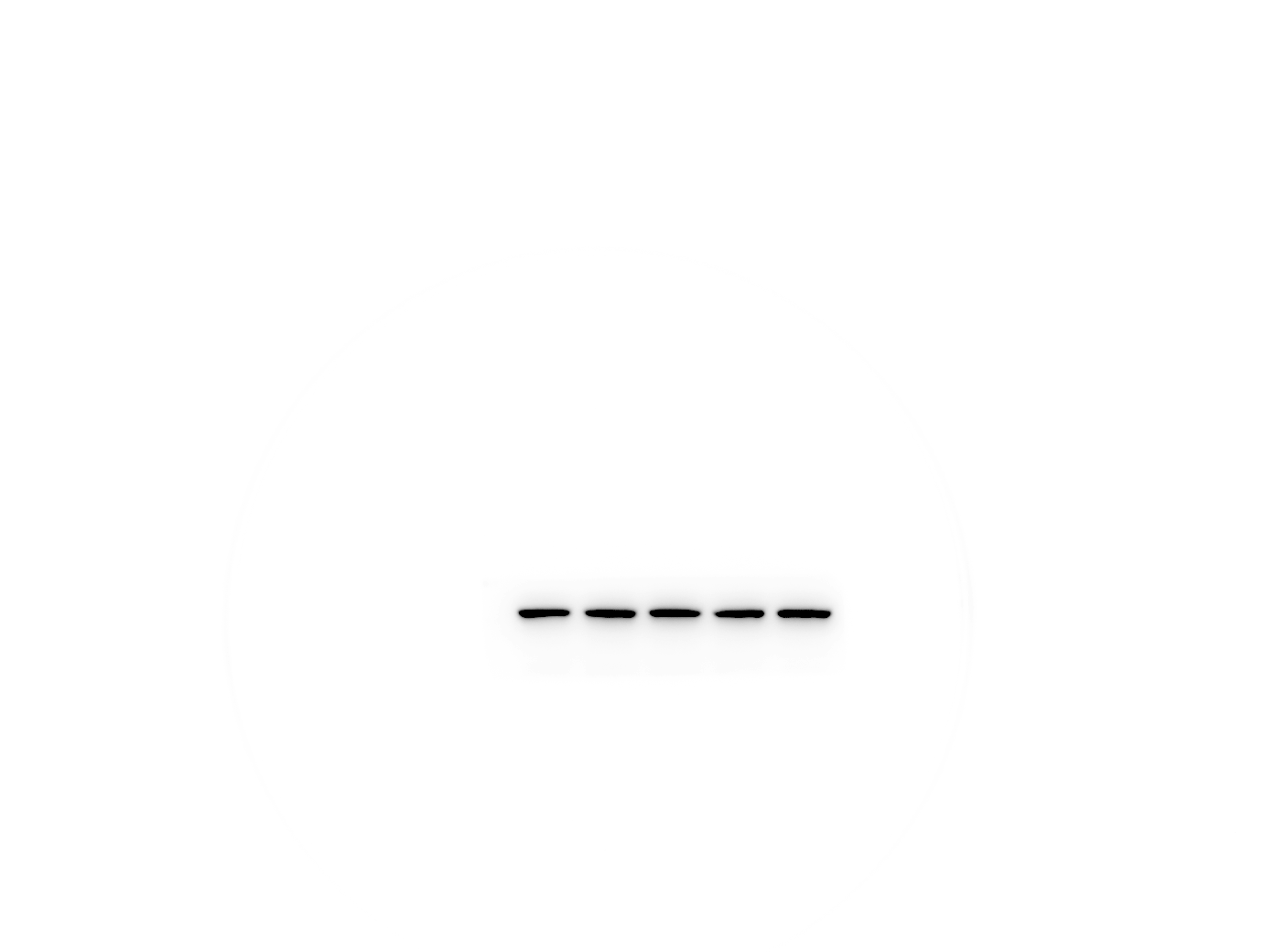


34

43

(kda) M CON Veh1 Veh2 Tre1 Tre2

Figure6-β-actin 42kda


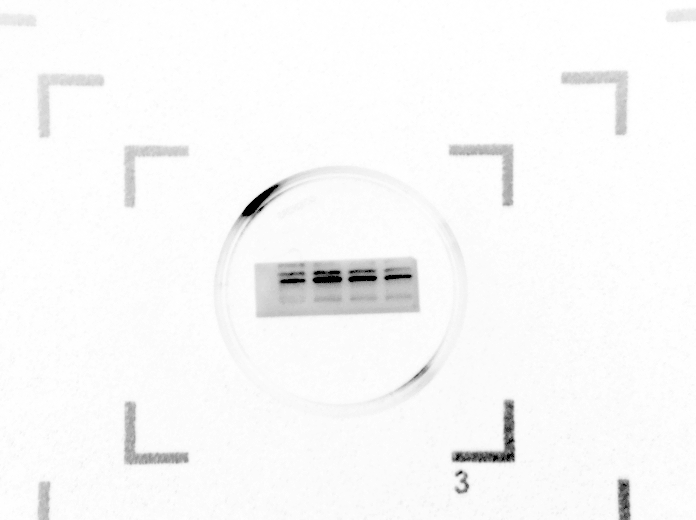


34

43

(kda) M CON OGD 0.1 1

Figure10-AIM2 43kda


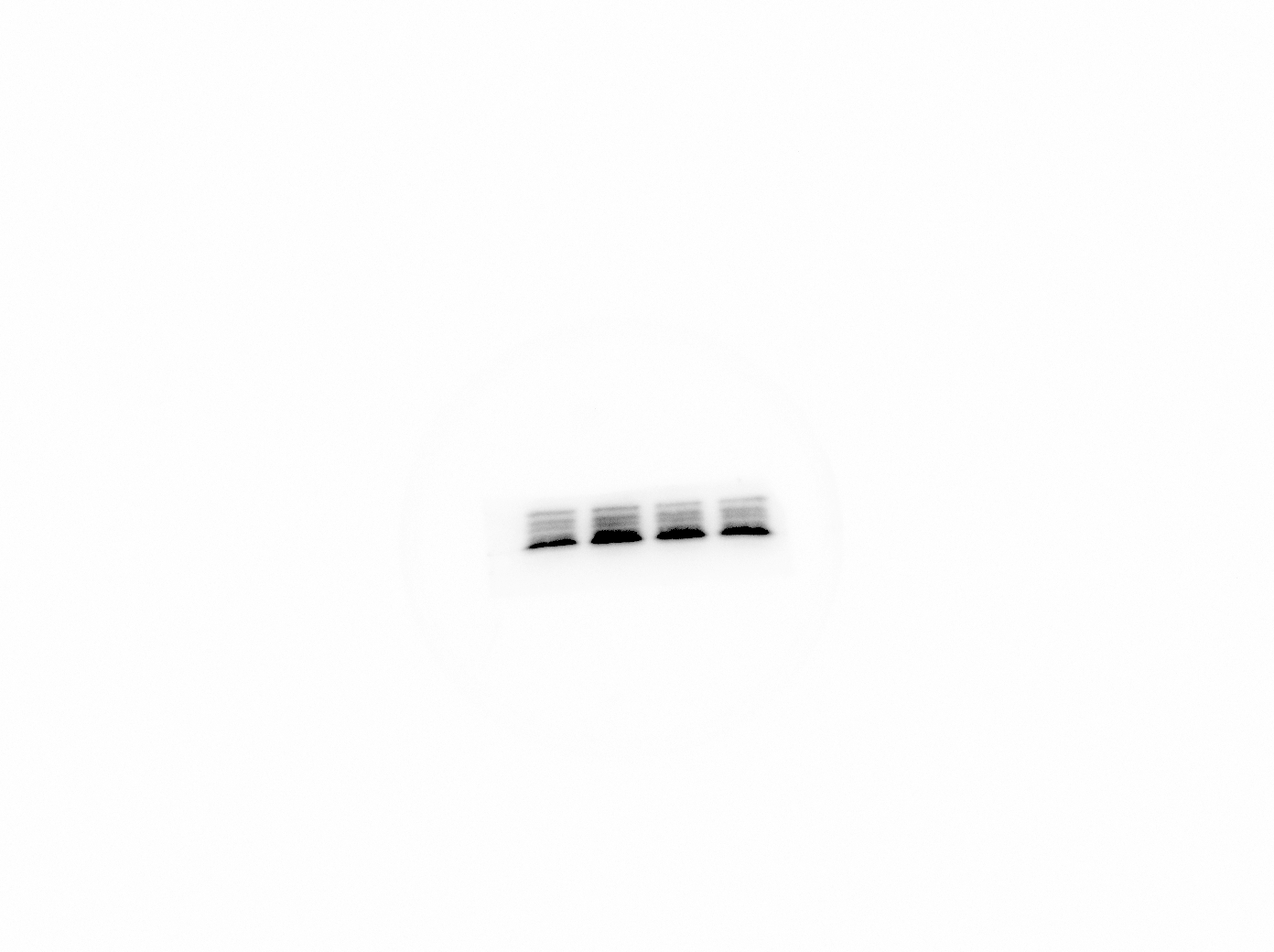


26

17

(kda) M CON OGD 0.1 1

Figure10-ASC 22kda


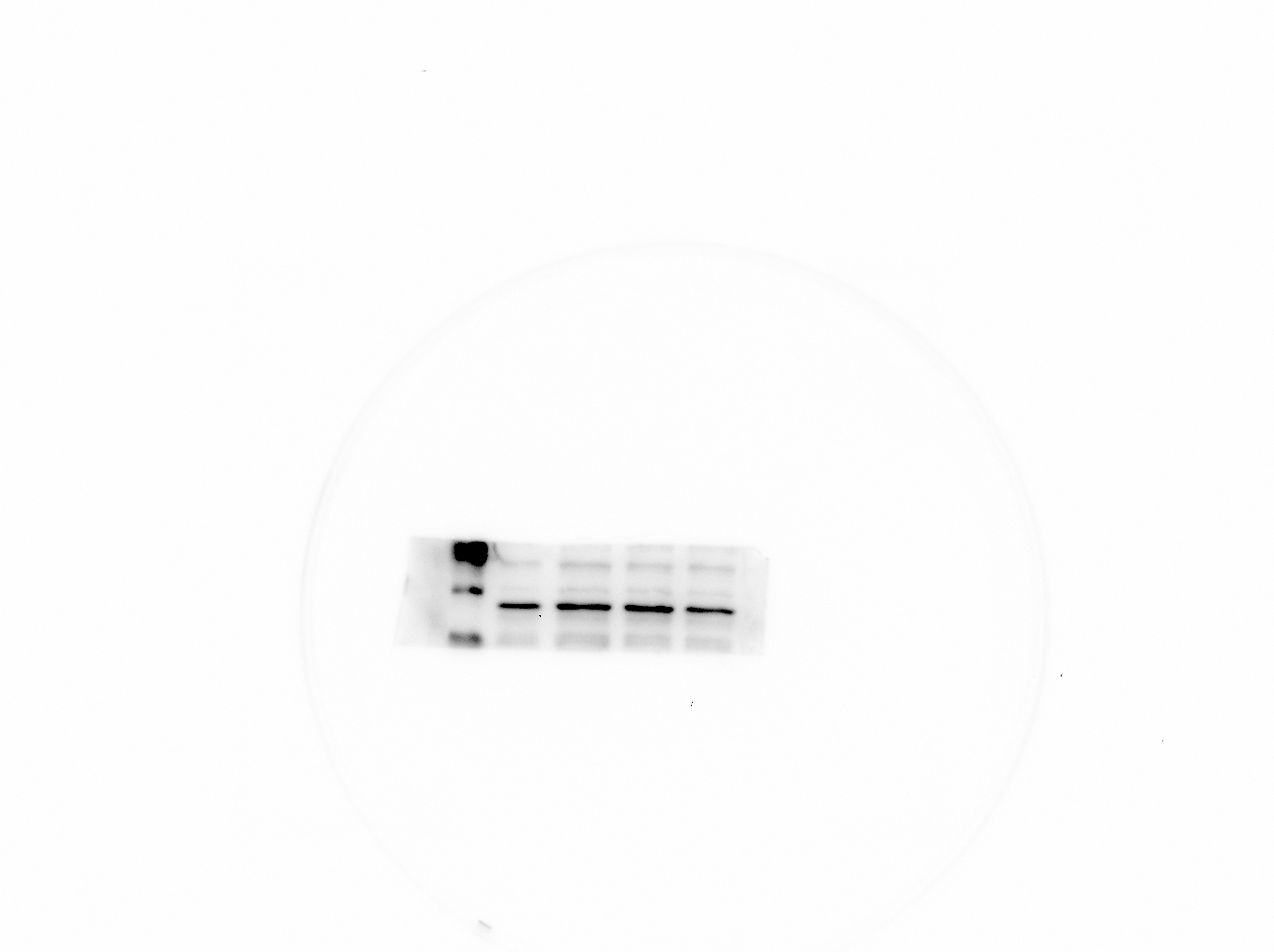


26

43

34

55

34

(kda) M CON OGD 0.1 1

Figure10-GSDMD 53,32kda


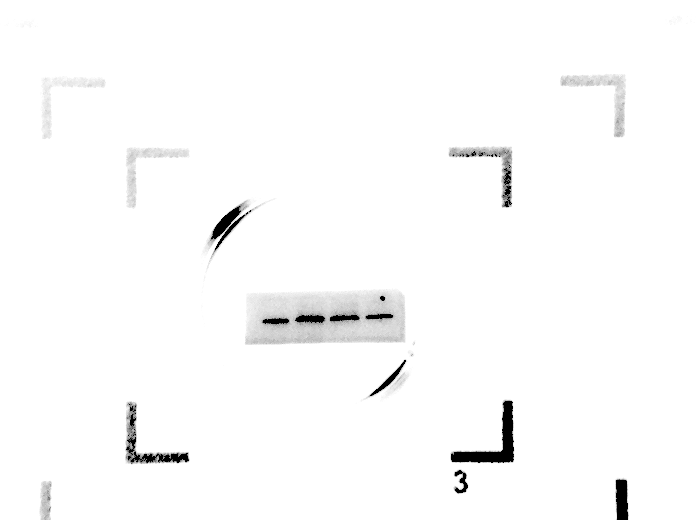


26

17

(kda) M CON OGD 0.1 1

Figure10-Cleaved-caspase1 22kda


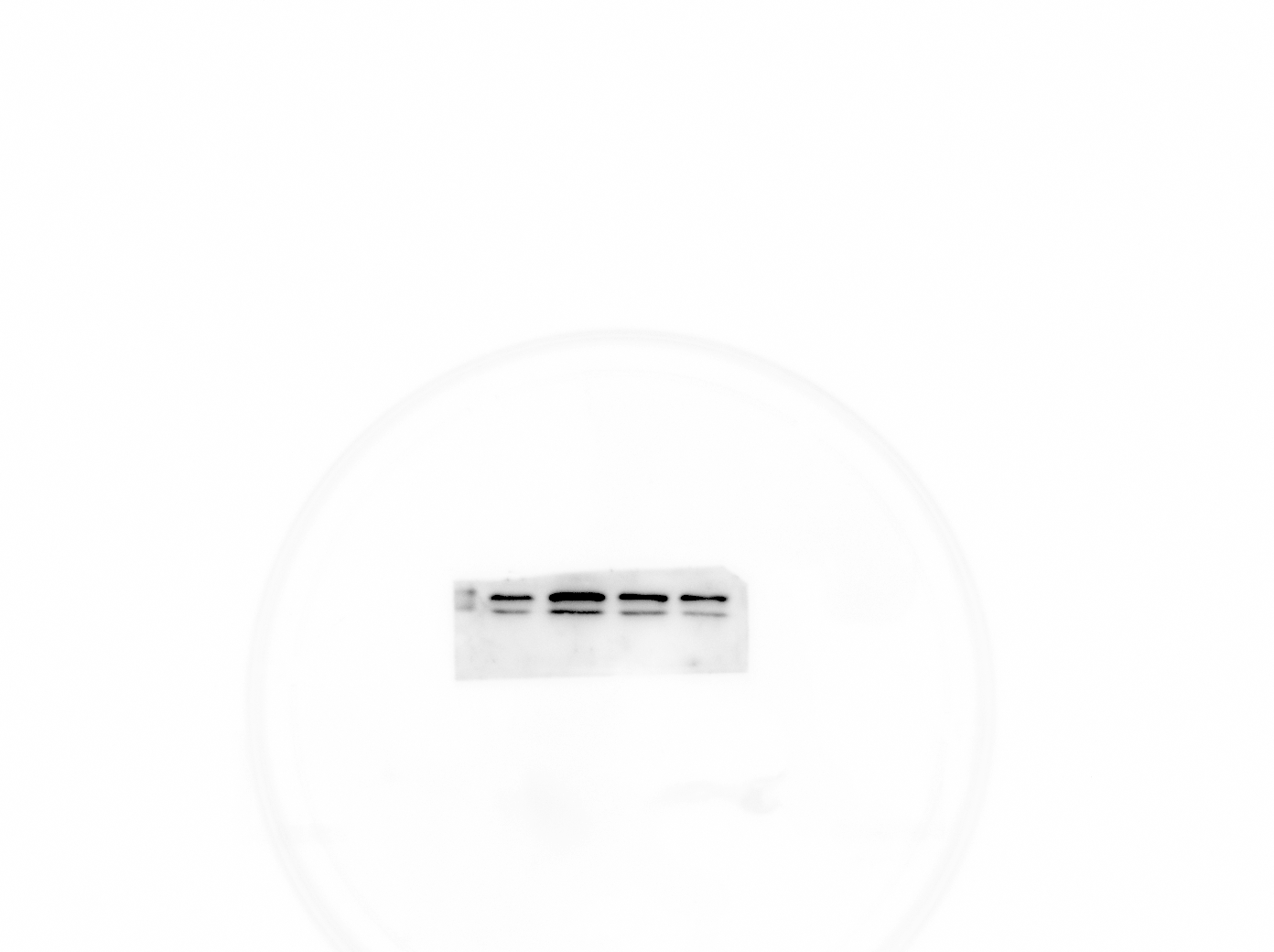


17

(kda) M CON OGD 0.1 1

26


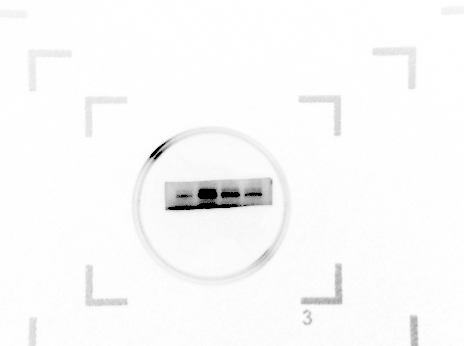


34

26

(kda) M CON OGD 0.1 1

Figure10-IL-1β 31kda


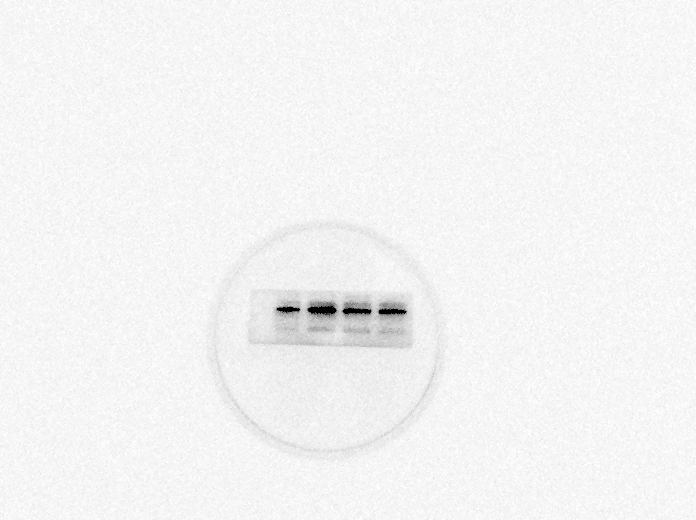


(kda) M CON OGD 0.1 1

55

43

Figure10-Caspase1 48kda


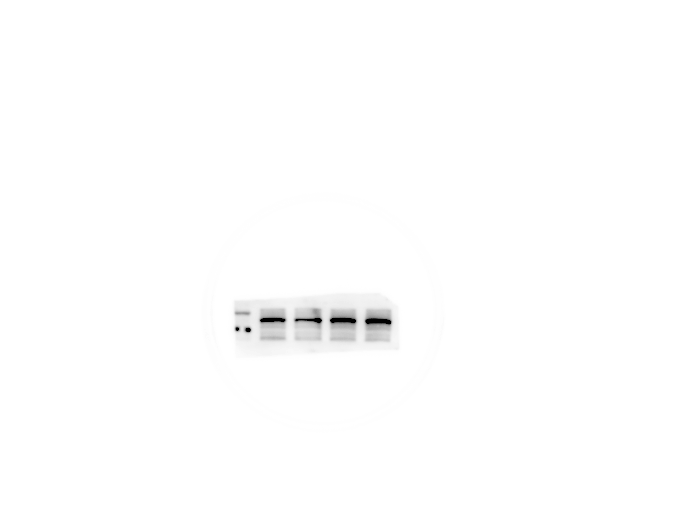


130

90

(kda) M CON OGD 0.1 1

Figure10-PGC-1α 100kda


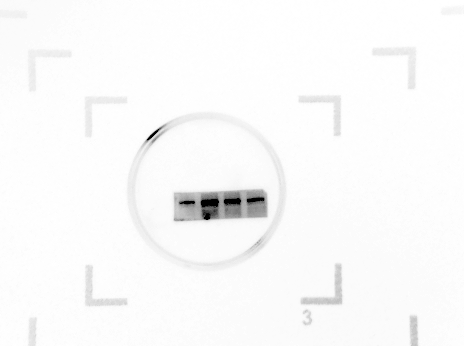


90

130

(kda) M CON OGD 0.1 1

Figure10-NLRP3 110kda


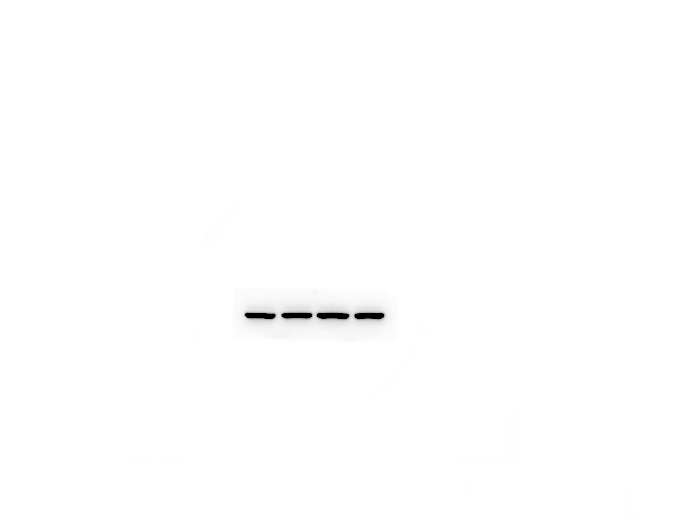


(kda) M CON OGD 0.1 1

43

34

Figure10-β-actin 42kda
